# A pulse of mid-Pleistocene rift volcanism in Ethiopia at the dawn of modern humans

**DOI:** 10.1038/ncomms13192

**Published:** 2016-10-18

**Authors:** William Hutchison, Raffaella Fusillo, David M. Pyle, Tamsin A. Mather, Jon D. Blundy, Juliet Biggs, Gezahegn Yirgu, Benjamin E. Cohen, Richard A. Brooker, Dan N. Barfod, Andrew T. Calvert

**Affiliations:** 1 Department of Earth Sciences, University of Oxford, South Parks Road, Oxford OX1 3AN, UK; 2 School of Earth Sciences, University of Bristol, Wills Memorial Building, Queens Road, Bristol BS8 1RJ, UK; 3 School of Earth Sciences, University of Addis Ababa, PO Box 1176, Addis Ababa, Ethiopia; 4 NERC Argon Isotope Facility, Scottish Universities Environmental Research Centre, Rankine Avenue, East Kilbride G75 0QF, UK; 5 U.S. Geological Survey, 345 Middlefield Road, MS-937, Menlo Park, California 94025, USA

## Abstract

The Ethiopian Rift Valley hosts the longest record of human co-existence with volcanoes on Earth, however, current understanding of the magnitude and timing of large explosive eruptions in this region is poor. Detailed records of volcanism are essential for interpreting the palaeoenvironments occupied by our hominin ancestors; and also for evaluating the volcanic hazards posed to the 10 million people currently living within this active rift zone. Here we use new geochronological evidence to suggest that a 200 km-long segment of rift experienced a major pulse of explosive volcanic activity between 320 and 170 ka. During this period, at least four distinct volcanic centres underwent large-volume (>10 km^3^) caldera-forming eruptions, and eruptive fluxes were elevated five times above the average eruption rate for the past 700 ka. We propose that such pulses of episodic silicic volcanism would have drastically remodelled landscapes and ecosystems occupied by early hominin populations.

## Abstract (web summary)

Past volcanic eruptions along the densely populated Ethiopian Rift valley remain poorly constrained despite the present day hazard. Hutchison *et al*. show that a large volcanic flare up along a 200 km section of the rift occurred between 320–170 ka dramatically affecting the landscape and hominin population.

## Graphical abstract

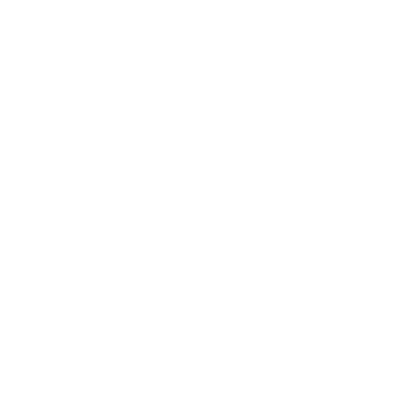

Geological records from many active and extinct continental rift zones reveal episodes of intense silicic volcanism, or ‘flare-ups' where eruption rates are elevated 3–10 times above the steady state (for example, ref. 1). Intracrustal magma reservoirs storing large volumes of evolved, eruptible magma are an essential prerequisite for these bursts of activity, and it is commonly assumed that such reservoirs can only be assembled when the mantle-to-crust magma flux is elevated significantly above the steady state^2^.

The 600 km-long Main Ethiopian Rift (MER) is a key sector of the East African Rift system connecting the Afar depression to the north, with the Turkana depression and Kenyan Rift to the south^3^. It is an active magmatic rift and hosts a current population in excess of 10 million^4^. Almost 40 years ago Mohr *et al*.^5^ speculated that a paroxysm of crustal extension and silicic volcanism (that is, a flare-up) took place in the MER during the Middle Pleistocene (between 300 and 200 ka). This hypothesis has important implications for the evolution of environments and habitats across and along the MER^6^, and for past, present and future volcanic hazards in the region. However, Mohr *et al*. had few constraints on ages or erupted volumes from individual silicic volcanic centres, and this flare-up hypothesis has not yet been thoroughly tested^6^.

The Middle Pleistocene (781–126 ka) of Ethiopia spans a key juncture in hominin evolution, represented by the arrival of anatomically modern humans (i.e., our species *Homo sapiens*) in the region at around 200 ka (ref. 7). Although the evolutionary stimulus at this time remains uncertain, current evidence overwhelming suggests that all major events in hominin evolution occurred in East Africa^8^. A recurrent theme of this research has been to link key hominin speciation, extinction and dispersal events to periods of environmental and/or climatic change^9^, and a particular emphasis has been placed on understanding glacial–interglacial climate fluctuations and the short alternating periods of arid–humid environmental variations this causes in East Africa. While attempts to understand the links between past environmental change and human evolution have stimulated considerable research into African palaeoclimate^10^, comparatively little attention has been paid to the role of explosive volcanism in influencing rift habitability^11^. The fundamental—and unanswered—questions are whether explosive volcanic activity in the MER has been steady or pulsed through the Quaternary; and whether eruptions, and the environmental disruption that followed, were of sufficient size to have caused significant dislocations in hominin populations and reduced their mobility along rift migration corridors.

In this paper, we present new age data for caldera-forming eruptions at Aluto and Corbetti volcanoes, as well as an analysis of eruption sizes along the rift, and use these to evaluate the Middle Pleistocene flare-up hypothesis^5^,^6^. We find that the onset of the Middle Stone Age in Ethiopia (ca. 300 ka; ref. 12) was indeed a period of elevated magmatic flux and caldera-forming volcanism. We consider the causes for the flare-up, the broader implications of this mode of activity, and the potential impacts both for early hominin populations living in the MER, and for current volcanic hazards in the region.

## Results

### Eruptive history of Aluto and Corbetti

**Figure 1:**
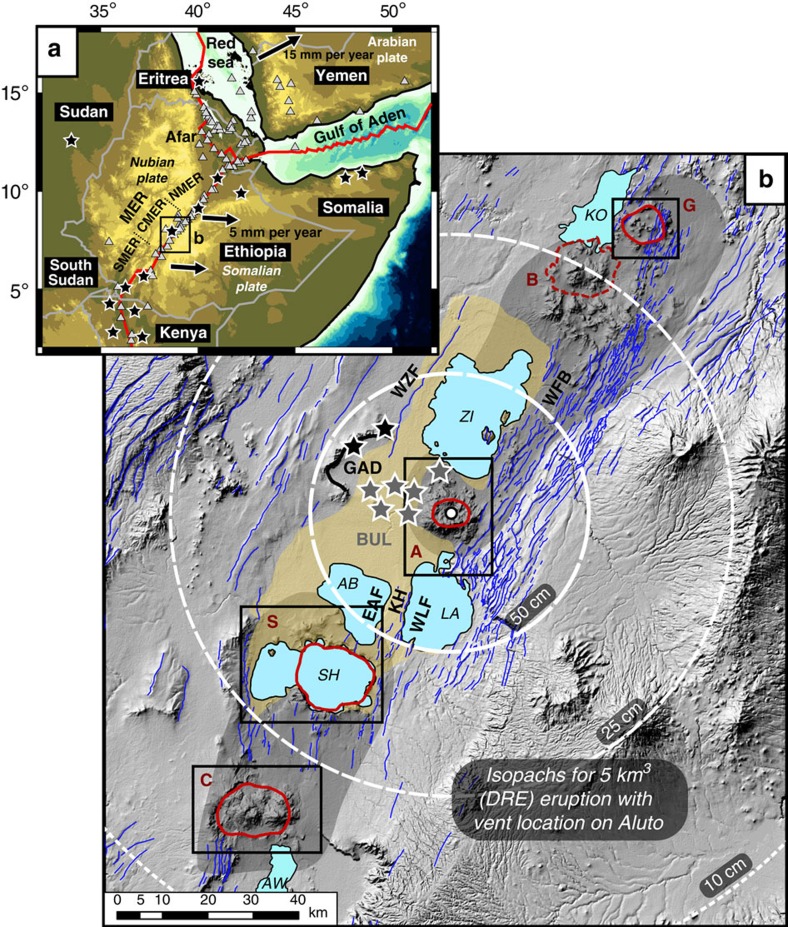
Topographic maps of East Africa and the central Main Ethiopian Rift. (**a**) Red lines indicate major plate boundaries, grey triangles show Quaternary volcanoes, black stars identify key archaeological sites dated as Middle Stone Age (MSA) (after ref. 11) and black arrows show current extension vectors (relative to a fixed Nubian plate^72^). The MER is divided into northern (NMER), central (CMER) and southern (SMER) segments after ref. 3. (**b**) Hillshade digital elevation model showing the study region. Quaternary silicic volcanic include the following: Corbetti (C); Shala (S); Aluto (A); Bora-Bericcio (B); and Gedemsa (G); solid red lines indicate calderas, dashed lines on Bora-Bericcio show approximate extent of silicic deposits. Black rectangles correspond to detailed geological maps in Fig. 2. Beige shaded area identifies the maximum extent of the Ziway–Shala lake system in the Late Pleistocene^15^. The major present-day lakes are identified as KO: Lake Koka; ZI: Lake Ziway; LA: Lake Langano; AB: Lake Abijata; SH: Lake Shala; and AW: Lake Awasa. Faults, after ref. 13, are shown as blue lines. Major structural features include the following: East Abijata fault (EAF); Katlo Horst (KH); West Langano fault (WLF); West Ziway fault (WZF); and the Wonji fault belt (WFB), summarized in Fig. 6 and by ref. 6. Approximate extent of the volcano-tectonic segments are shown by black shaded areas^6^,^44^. Black stars show MSA sites at Gademotta Ridge (GAD), grey stars show Late Stone Age sites adjacent to the Bulbula River (BUL). Illustrative isopachs (thickness of tephra fall deposits) predicted for an eruption of 5 km^3^ silicic magma (equivalent to 12 km^3^ of tephra), were calculated using the exponential thinning relationship^64^ and are shown as white circles (the vent location is centred on Aluto, for simplicity no external wind field was considered, and we have assumed a conservative tephra thickness of 1 m proximal to the vent).

**Figure 2:**
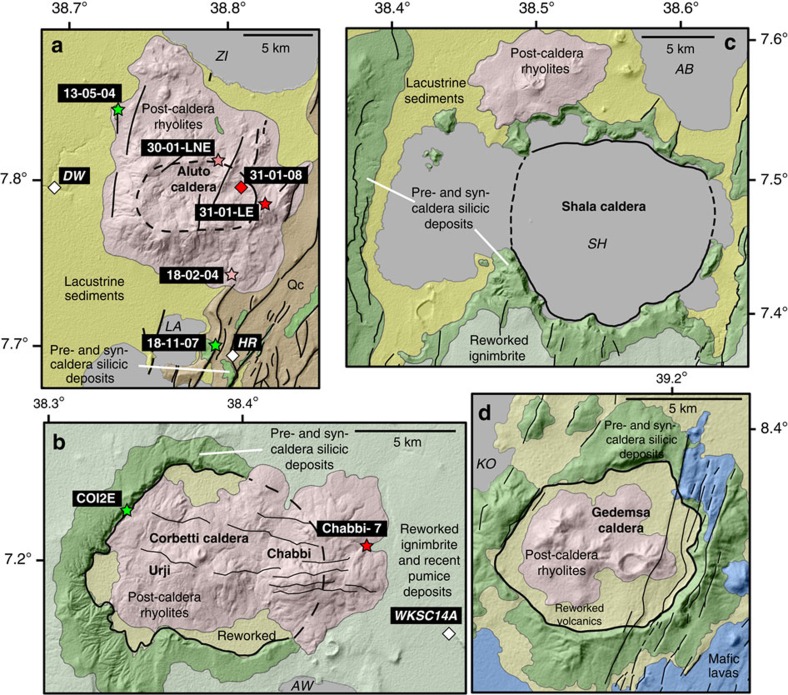
Geological maps of the silicic volcanic complexes discussed in this study. The geology of Aluto (**a**) and Corbetti (**b**) was constrained by our new field mapping and geochronology (Fig. 3), as well as refs 16, 17 while the geology of Shala (**c**) and Gedemsa (**d**) is primarily based on previous mapping by refs 5, 24. Units sampled for ^40^Ar/^39^Ar geochronology are shown by the stars, while radiocarbon samples are shown by the diamonds (white colours represent ages determined by previous authors while coloured symbols represent our new age determinations, see Table 1 for details). The caption beside each symbol refers to the sample code except in the case of Deka Wede (DW) and Haroresa (HR), where multiple radiocarbon ages were determined at a single stratigraphic section (a full list of sample codes and localities is provided in Table 1). Each volcano underwent a period of edifice building before caldera collapse and we combine these deposits, which are exposed in caldera wall or faulted sections, as pre- and syn-caldera silicic deposits (dark green). Post-caldera silicic volcanic products (predominantly represented by obsidian coulees and pyroclastic density current deposits) are shown in pink colours. At Aluto the post-caldera volcanism has covered almost all of the original caldera structure^16^. Other simplified geological units include the following: lacustrine sediments (late Pleistocene-Holocene deposited in the Ziway–Shala basin^15^,^19^); reworked volcanic sediments; mafic (Wonji) lavas; and alluvial–colluvial sediments deposited on the rift margin (unit Qc, east of Aluto). The major present-day lakes are identified as KO: Lake Koka; ZI: Lake Ziway; LA: Lake Langano; AB: Lake Abijata; SH: Lake Shala; and AW: Lake Awasa.

**Figure 3:**
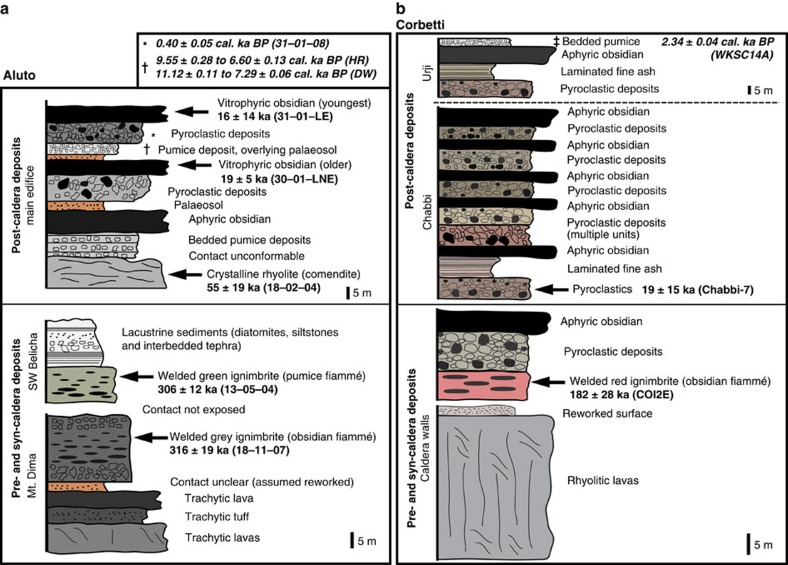
Simplified eruptive stratigraphy for Aluto and Corbetti volcanoes. Aluto is shown in **a** and Corbetti is shown in **b**. Arrows indicate units with new ^40^Ar/^39^Ar ages (mean±one s.d.) while symbols (*, † and ‡) indicate radiocarbon ages (mean±s.d. of the calibrated range at the 95.4% probability range). Radiocarbon ages represented by † bracket eruptive deposits, while the others represent material dated from beneath the volcanic deposit. Sample localities and relevant codes are shown on the geological maps in Fig. 2.

Aluto and Corbetti are two major silicic volcanoes located in the central MER (CMER^3^,^13^,^14^, Fig. 1a). Aluto is situated within the Ziway–Shala basin (Fig. 1b), a tectonically controlled drainage basin that formed a single connected lake in the Late Pleistocene (∼100 ka)^6^,^15^, while Corbetti is located at the northern end of the Lake Awasa basin. New geological maps for Aluto and Corbetti are presented in Fig. 2a,b, respectively. Both complexes have caldera structures, thought to have formed in response to large explosive eruptions that deposited extensive ignimbrite sheets^16^,^17^. We established the volcanic stratigraphy of Aluto and Corbetti during field campaigns, and collected unweathered rock and charcoal samples from the sites shown in Fig. 2 (coloured stars and diamonds) for geochronological analysis. New ^40^Ar/^39^Ar and radiocarbon ages were used to verify our proposed volcanic stratigraphies and are presented in Fig. 3 and Table 1.

Full details of our geochronological analyses are provided in the Methods section. For consistency throughout this article, and taking precedent from existing hominin literature (for example, ref. 7), all ^40^Ar/^39^Ar ages represent the arithmetic mean and the error quoted is one s.d. of the data. This choice of error reporting does tend to inflate the errors on the youngest post-caldera samples from Aluto and Corbetti, and so in Table 1 we also report the ages as weighted mean±s.e.m. This is an equally valid format for age reporting, and the error magnitudes are typical of very young Quaternary deposits.

The deep stratigraphy of Aluto is exposed along faults on the margins of the complex (Fig. 2a). South-east of the main edifice (Fig. 2a), a sequence of massive, dull-grey, strongly welded ignimbrites, rich in lithic fragments and with abundant obsidian fiammé, are exposed (Fig. 3). This major grey welded ignimbrite sequence is typically 20–50 m thick and is underlain by trachytic lavas, breccias and minor tuff horizons that comprise the earliest silicic eruptions of Aluto (Fig. 3). Our interpretation is that these underlying trachytic lava flows and tuffs, which have a minimum thickness of 100 m, represent a low relief pre-caldera edifice (or silicic lava shield) that was built up on the faulted rift terrain.

Massive, pistachio green, welded ignimbrites are also exposed west of Aluto (Fig. 2), here containing abundant pumice fiammé. The deposit is immediately overlain by lacustrine sediments (Fig. 3) suggesting that it was emplaced in a lake basin. New ^40^Ar/^39^Ar geochronology (Fig. 3) reveal that the Aluto grey and green welded ignimbrites have overlapping ages of 316±19 and 306±12 ka, respectively. While there is insufficient field evidence to assess how these deposits physically relate, their componentry (fiammé, >10 cm diameter), thicknesses (>10 m) and dense welding indicate that they are the proximal deposits of large-scale ignimbrite-forming eruptions (>>1 km^3^).

The main edifice of Aluto (Fig. 2) comprises a pile of coalescing obsidian coulees and pumiceous pyroclastic deposits. Comenditic rhyolites with ^40^Ar/^39^Ar ages of ∼60 ka were identified at the base of the edifice, while the youngest obsidian coulees have ^40^Ar/^39^Ar ages of 10–20 ka (Fig. 3 and Table 1). A substantial data set of radiocarbon ages has been established for a sequence of tephra and interbedded lacustrine sediments exposed along the Bulbula river ∼10 km west of the Aluto edifice^6^,^15^,^18^ (BUL in Fig. 1b). The largest volcaniclastic sequence, informally known as the Abernosa pumice, was deposited between ∼26 and ∼14 calibrated ka BP, and preserves ∼13 distinct tephra layers^18^. The youngest widely dispersed tephra layer that has been identified in lacustrine sections west^6^,^15^,^18^ and south-east^19^ of Aluto shows overlapping ages between ∼11 and ∼7 cal. ka BP (Table 1). Finally, a new radiocarbon age beneath a thin bed of pumiceous pyroclastic deposits (20–30 cm thick) collected on the west of the Aluto edifice (Fig. 2) reveals that the youngest explosive eruptions on the complex took place around 0.4 cal. ka BP. The new and existing radiocarbon ages, summarized in Table 1, provide clear evidence for abundant explosive volcanism at Aluto between 0 and 30 ka. Although the radiocarbon and ^40^Ar/^39^Ar ages reflect different volcanic processes and hence are not directly comparable, they do show good overlap and demonstrate that post-caldera activity at Aluto has taken place between 0 and 60 ka.

At Corbetti, the earliest activity is represented by a sequence of rhyolitic lava flows and domes (>50 m thick) exposed in the caldera walls (Fig. 2). The pre-caldera lavas are covered by massive, red, welded ignimbrites that yielded a ^40^Ar/^39^Ar age of 182±28 ka (Fig. 3). Unwelded pyroclastic deposits and aphyric obsidian coulees overlie the welded ignimbrite; together this sequence represents a single major eruptive unit and is the largest explosive eruption to have occurred at the Corbetti complex.

Following the major ignimbrite eruption, two eruptive centres (Chabbi and Urji, Fig. 2) developed within the collapsed caldera^17^. Post-caldera activity at these centres is characterized by a succession of explosive and effusive rhyolites, predominantly pyroclastic flows and aphyric obsidian coulees (Fig. 3). The oldest deposits of these cones have a ^40^Ar/^39^Ar age of ∼20 ka (Fig. 3 and Table 1). The youngest widely dispersed tephra from Corbetti, the Wendo Koshe Pumice, was erupted shortly after ∼2.3 cal. ka BP (ref. 17) and provides a robust radiocarbon age for the young post-caldera volcanism.

**Figure 4:**
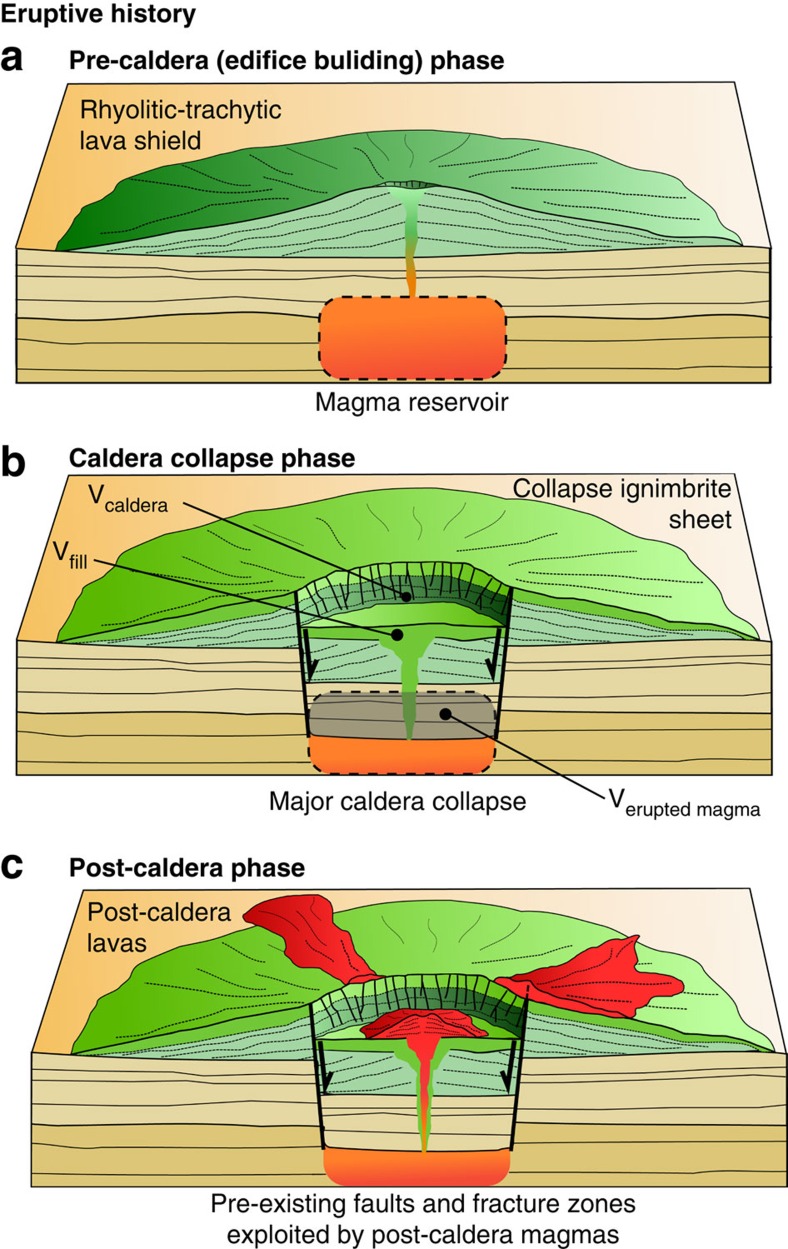
Schematic showing the simplified evolutionary history for silicic caldera complexes considered in this study. This three-stage evolution is a reasonable approximation of the long-term eruptive cycle at silicic peralkaline volcanoes^25^ and is also consistent with our geological mapping (Fig. 2) and the observations of previous authors^5^,^17^,^24^. At each stage the erupted volumes are estimated as follows, for **a** we assume that the pre-caldera volcano is a simple conical structure, and by measuring the slope of the edifice away from the central vent (perpendicular to caldera walls) and using thickness measurements of pre-caldera deposits measured in caldera walls and faulted sections volume can be calculated. For **b** we assume a piston style caldera collapse, and that the volume (*V*) of erupted magma approximates the volume of the caldera plus the volume of the intra-caldera fill (that is, *V*_erupted magma_≈*V*_caldera_+*V*_fill_). Deep-well ignimbrite thicknesses on Aluto are used to evaluate typical thicknesses of intracaldera fill. For **c** we mask post-caldera deposits from a present-day DEM, derive a smoothed pre-eruption surface and subtract the present-day DEM from the interpolated DEM to provide an estimate of erupted volume. Further details are provided in the Methods sections, and additional information on erupted volume constraints at each volcanic complex are provided in Supplementary Tables 1–3.

The new chronostratigraphic results support a simple staged eruptive history for both Aluto and Corbetti volcanoes (summarized in Fig. 4). Each underwent an early phase of silicic activity, building a low relief shield, as observed at other peralkaline volcanoes throughout East Africa (for example, Kone^20^, Menegai^21^,^22^, Olkaria^23^ and Longonot^23^). The pre-caldera activity was followed by a major phase of caldera collapse, and ignimbrite emplacement, and later post-caldera volcanism progressively in-filled the calderas^16^. At both Aluto and Corbetti the youngest post-caldera eruptions have taken place within the last 100–1,000 a (years), underscoring that these remain active volcanic complexes (Fig. 4).

### Wider evidence for a flare-up

Shala and Gedemsa volcanoes^5^,^24^ (S and G in Fig. 1b), show similar patterns of edifice growth, collapse and post-caldera resurgence, and can be compared directly with Aluto and Corbetti via the new geological maps in Fig. 2. At Shala, caldera collapse at 240±30 ka (K/Ar dating, after ref. 5) followed a period of rhyolitic–trachytic edifice growth, and eruption of pumiceous and welded ignimbrites^5^. At Gedemsa, north-east of the Ziway–Shala basin (Fig. 1b), the largest ignimbrite eruptions and resultant caldera collapse also followed a phase of silicic edifice building represented by rhyolitic lavas, pumice fall and ignimbrite deposits^24^. K/Ar dating^24^ showed that pre- and post-caldera rocks bracket an age of 320–260 ka for collapse events at Gedemsa.

Evidence from Gedemsa, Aluto, Shala and Corbetti volcanoes points to a common eruptive history in three stages: early edifice growth (pre-caldera phase); caldera collapse; and a post-caldera phase (summarized in Fig. 4 with additional details in Supplementary Tables 1–3). These stages are a reasonable approximation of the long-term eruptive cycle at silicic peralkaline volcanoes^25^, and our geochronological constraints are sufficiently well resolved to establish when the transitions between these stages occurred. It is important to clarify that, with the exception of Bora-Bericcio volcano (Fig. 1b), which has yet to be studied in any detail, existing geochronological evidence suggests that all silicic volcanism within our study region (Fig. 1b) and period of interest (i.e., the last 700 ka) took place at the complexes considered here (Aluto, Corbetti, Shala and Gedemsa). Previous work on silicic volcanism in the MER has predominantly focused on the Pliocene and has shown that several major silicic calderas (for example, Munesa, Awassa and Gademotta^26^,^27^) were active along the axial region of the CMER between 4 and 1 Ma, these complexes are now extinct and deeply eroded or otherwise buried by sediments. Further, the trachytic volcanoes that occur on the Ethiopian Highland (for example, Chilalo, Kaka, Hunkulu and the Bada Range) also display K/Ar ages from 3.5 to 1.0 Ma (refs 26, 28). None of these volcanoes were active during the Middle Pleistocene (781–126 ka), and in the context of our study timeframe we underscore that they did not contribute to the silicic eruptive flux.

In Fig. 4 we describe a methodology for estimating eruptive volumes at each stage using digital elevation models (DEMs) and deposit thicknesses. Our approach is described in detail in the Methods section and for clarity we briefly state our key assumptions. First, we assume that the pre-caldera edifice is represented by a simple conical construct and that edifice growth began 250 ka before the caldera-forming eruption (in line with geochronological observations from other peralkaline volcanoes in East Africa^21^,^23^,^29^,^30^). For the caldera collapse phase, we approximate the structure as a cylindrical subsiding block^31^ and use the volume of the caldera, plus intracaldera fill^32^, to estimate the volume of material ejected by the eruption^33^. Field evidence (Fig. 3 and refs 5, 24) suggests that the collapse ignimbrites were deposited without major hiatuses and can be considered as geologically instantaneous. Finally, for the post-caldera phase we use DEMs to mask out these volcanic deposits, calculate a smoothed pre-eruption DEM surface, and use the residual volume between the present day and interpolated DEM surfaces to calculate erupted volumes^34^.

**Figure 5:**
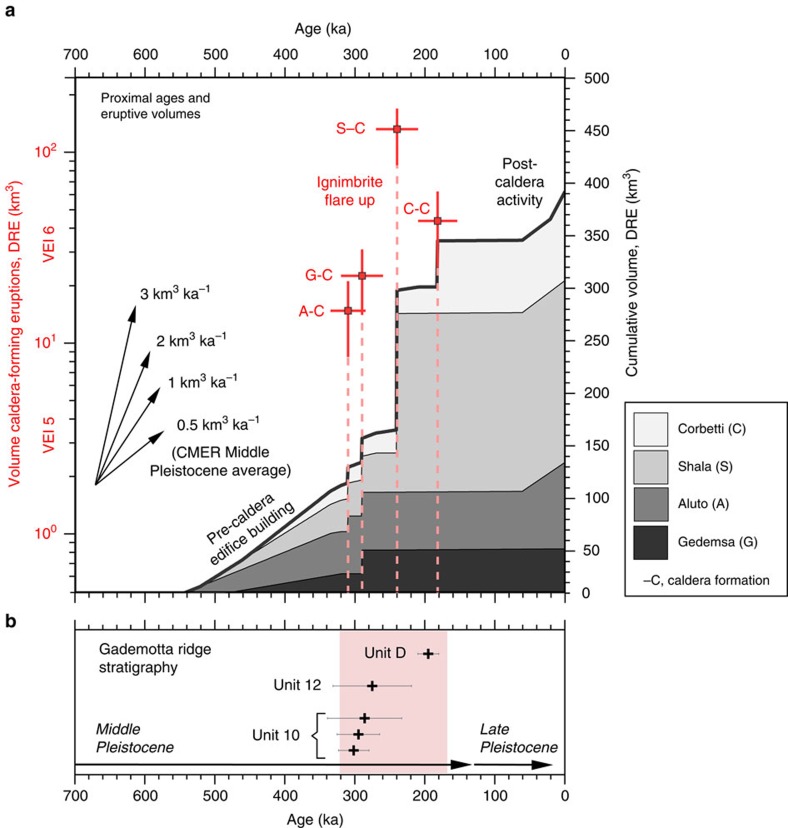
Timescales of silicic volcanism in the central Main Ethiopian Rift during the Pleistocene. (**a**) Age–volume plot for Gedemsa (G), Aluto (A), Shala (S) and Corbetti (C) volcanoes. The left-hand axis shows the volume of individual caldera-forming eruptions (log scale) and corresponds to the dashed red bars (labelled for each volcano). The volcanic explosivity index (VEI) of each eruption is also indicated on the left-hand axis. Red data points indicate the timing of caldera formation (i.e., the mean age of the caldera eruption) and eruptive volume estimate (see Supplementary Table 2 for full details), horizontal error bars show the s.d. of the age data (except in the case of Gedemsa when caldera formation is bracketed by dates), vertical error bars show the low and high estimates for caldera eruption volume (Supplementary Table 2). The right-hand axis shows the cumulative volume for the complexes (individual volcano records are stacked). For the pre- and post-caldera phases we have assumed linear magma supply rates. Arrows show eruptive fluxes of 0.5, 1, 2 and 3 km^3^ per ka. Peak eruptive flux occurs between 320–170 ka when the major caldera-forming events occurred at all four volcanoes. Between 320 and 170 ka the silicic eruptive flux was 2.5 km^3^ per ka; five times greater than the time-averaged Middle Pleistocene (781–126 ka) eruption flux of 0.5 km^3^ per ka, and approaching long-term magmatic output from Taupo Volcanic Zone and Yellowstone (∼3 km^3^ per ka)^35^. (**b**) ^40^Ar/^39^Ar ages for tephras from Gademotta Ridge^12^,^37^,^38^ (ages recalculated to decay constants of ref. 54 where necessary). Black horizontal bars give typical 1*σ* s.e.m. for each tephra as originally quoted by refs 37, 38. Grey horizontal bars show one s.d. of the data (i.e., equivalent to the error quoted on our new ^40^Ar/^39^Ar ages throughout the manuscript).

The age-erupted volume plot is shown in Fig. 5a. The cumulative erupted volume is dominated by Shala caldera collapse, although caldera-forming eruptions at the other silicic volcanoes also contributed significant pulses of silicic magma. From these data we estimate the Mid-Pleistocene (781–126 ka) average eruption rate to be 0.5 km^3^ per ka. From 320 to 170 ka the eruption rate is 2.5 km^3^ per ka, five times the Mid-Pleistocene average, and comparable to the long-term magmatic output from Taupo Volcanic Zone and Yellowstone (∼3 km^3^ per ka, ref. 35), the largest active silicic magmatic systems on Earth.

Further evidence for the flare-up is found at Gademotta Ridge, ∼20 km north-west of Aluto (GAD in Fig. 1b), where colluvial–alluvial sediments and tephra preserve hominin occupation sites from the Middle and Late Pleistocene^12^,^36^,^37^,^38^. Three major distal tephra layers have been identified at Gademotta Ridge, they are 0.5–1 m thick and of fallout origin^36^. ^40^Ar/^39^Ar ages^12^,^37^,^38^ for the tephra units range from 302±22 to 195±15 ka (Fig. 5b, overlapping the flare-up period). The Gademotta stratigraphy represents an independent record of Pleistocene volcanism and not only testifies that several major explosive eruptions occurred during the flare-up period, but that significant volumes of tephra were dispersed across and along the rift (Fig. 5).

We have shown that four silicic volcanic complexes along a ∼200 km segment of the MER all underwent their largest explosive eruptions in a geologically short time span between 320 and 170 ka (Fig. 5a). It is also important to recognize that major phases of edifice construction were also taking place during the flare-up period, as evidenced by existing ages from Shala^5^ and Gedemsa^24^, 280±10 and ∼320 ka, respectively, strengthening the case for a major pulse of silicic volcanism at this time. It is unlikely that the magmatic reservoirs of the flare-up ignimbrites were connected. The ∼40 km distance separation between the complexes, lack of nested collapse structures (Fig. 1b), as well as geophysical imaging of silicic systems from elsewhere in the MER^39^, all suggest that the complexes host discrete shallow magmatic reservoirs. Treating the caldera-forming eruptions as independent, and assuming each major eruption could have occurred randomly during the ca. 500 ka ‘life-time' of these volcanoes then the cumulative Poisson probability that more than three eruptions occurred in a 150 ka window (pink shaded area in Fig. 5b) would be 0.03. This underscores that a chance clustering is highly unlikely.

## Discussion

The new geochronological constraints and volume estimates provide the first robust evidence for flare-up activities in the CMER between 320 and 170 ka, and support the hypothesis of Mohr *et al*.^5^ who first proposed a pulse of volcanism at this time. Flare-ups are often linked to periods of increased mantle magma productivity that elevate mantle-to-crust magmatic fluxes significantly above the steady state^2^. During these episodes of increased magmatic and thermal input to the crust, large volumes of chemically evolved, eruptible silicic magma are generated^40^. Once assembled, eruption of spatially separated, melt- and volatile-rich reservoirs are triggered internally by magmatic overpressure, or through external processes such as regional stress changes and faulting^41^. We briefly outline the spatial and temporal constraints on the flare-up and develop a hypothesis surrounding its origins.

**Figure 6:**
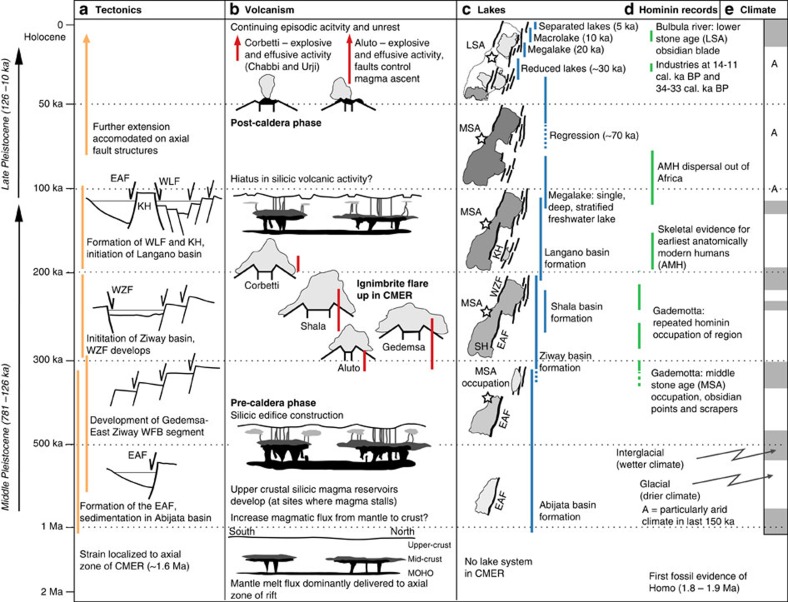
Synthesis of the tectonic, volcanic, lake, climate and hominin records for the Ziway–Shala rift basin. Coloured vertical bars for (**a**) tectonic (orange), (**b**) volcanic (red), (**c**) lacustrine (dark blue) and (**d**) hominin records (green) represent the existing age uncertainties from this study and refs 6, 15, 37, 73. Simplified records of (**e**) climate are shown by Pleistocene glacial–interglacial cycles, shaded white and grey, respectively, which are summarized from stacked benthic foram δ^18^O time series^74^. Major structural (**a**, tectonic) features, including the East Abijata fault (EAF), the Katlo Horst (KH), the West Langano fault (WLF), the West Ziway fault (WZF) and the Wonji fault belt (WFB), are shown on Fig. 1b, and summarized after ref. 6. Shading of the lakes gives a qualitative representation of their depth (darker colour indicates deeper water). Stars identify hominin occupations such as the Middle Stone Age (MSA) sites of Gademotta Ridge (GAD, Fig. 1b) and Late Stone Age (LSA) sites adjacent to the Bulbula River (BUL, Fig. 1b). The development and changing habitability of the Ziway–Shala basin represents a complex interplay between tectonic, volcanic and climatic processes; a detailed understanding of each of these processes and their potential coupling is essential for fully appreciating the records of hominin evolution and migration in the region.

The record of volcanism in the CMER from the middle-Pleistocene to recent (Fig. 5a) is consistent with a ‘pulsed model' of silicic magma productivity characterized by episodes of major ignimbrite eruptions (320–170 ka), but also by significant hiatuses (for example, the post-caldera phase, where >150 ka elapsed before volcanism resumed at Aluto and Corbetti, and only very small volumes, <5 km^3^ dense rock equivalent (DRE), have been erupted at Gedemsa and Shala). Temporal clustering of the ignimbrite eruptions (Fig. 5a) suggests that development of the silicic magma reservoirs was closely synchronous. Thermal and density considerations require that large upper-crustal magma bodies (that is, flare-up magmas) are geologically short-lived^42^, and therefore the clustering of eruptions implies that assembly of the separate silicic magma reservoirs was approximately coeval. To initiate simultaneous silicic magma reservoir development along the rift we suggest that a period of elevated mantle-to-crust magma flux is necessary (Fig. 6), caused by an increase in mantle melt production and/or change in rift architecture and kinematics that promoted increased magmatic intrusion of the continental lithosphere (see ref. 14 for detailed discussion on mechanisms).

Melt flux out of the MER mantle reservoir would be focused into axial volcano-tectonic segments; <20 km wide, ∼50 km long, en-echelon features located along the axis of the rift^3^,^14^. The volcano-tectonic segments in the CMER have been active since ∼1.6 Ma (ref. 6 and Fig. 6), and there is evidence that large volumes of mafic magma are intruded into the roots of the segments (up to depths of ∼10 km)^43^. The silicic volcanoes are located at the ends of the volcano-tectonic segments^5^,^43^,^44^ (Fig. 1b), presumably where magma ascent was hindered due to the complex extensional stress field^43^ and/or the cooled crust abutting the segment tip^45^ allowing stalling, chemical differentiation and accumulation of silicic melts in the upper crust.

Synthesizing these concepts, we propose that an increased melt flux out of the mantle reservoir, delivered to the axial volcano-tectonic segments of the MER initiated growth of the flare-up magmas, and that melt focusing, by capture^46^ and/or stalling at the segment tips^43^,^45^, provided an important structural trap. Although deep stratigraphic exposures of the pre-flare-up period are limited within the rift, at Aluto evidence from geothermal wells reveals that before the first silicic eruptions, voluminous mafic fissure eruptions occurred at ∼1.6 Ma generating ∼1 km-thick sequences^47^. We suggest that the magma bodies for the flare-up ignimbrites represent the stalled, structurally focused, component of this large mantle-derived melt flux (Fig. 6). Crucially, the timescales are consistent with thermal–petrological models of continental rifts^48^ that require 1–2 Ma of mafic magma injection before evolved melt-mush zones develop within the upper crust.

Once large silicic magma reservoirs were assembled ignimbrite eruptions could be initiated. The precise trigger of the flare-up between 320 and 170 ka does however remain open to debate. Either, the major eruptions were driven by processes internal to the silicic magma bodies and the separate reservoirs each reached critical overpressure, or alternatively, eruptions were triggered by external processes such as fault growth in the Ziway–Shala basin^5^,^6^ (Fig. 6), static stress changes in response to neighbouring caldera collapses or dyke intrusions. Detailed geochemical investigation of the flare-up magma reservoirs, in terms of crystallization, thermal and storage histories^2^,^41^, will be central to testing these hypotheses.

Environmental change and variability have been proposed as drivers for the major events in East African hominin evolution^8^. Recent studies have tended to focus on glacial–interglacial climate fluctuations, leading to short alternating periods of arid–humid environmental variations in East Africa, as the main control on human evolutionary and cultural development^9^,^49^. We suggest that a fuller understanding of the drivers of environmental change and its consequences in an active rift setting requires a broader perspective.

In Fig. 6, we synthesize the Quaternary history of the Ziway–Shala rift basin using the detailed tectonic and climatic records developed previously. Localization of faulting along the rift axis of the CMER began by ∼1.6 Ma (ref. 6) and led to the development of the volcano-tectonic segments (Fig. 1b). This overlaps with the age of the pre-Aluto mafic lavas, 1.6±0.5 Ma (ref. 47), that are encountered in the deep wells beneath the complex. Taking into consideration this age and its uncertainty, as well as the considerable thickness 500–1,000 m of largely uninterrupted mafic lavas flows and scoria suggests that there was a significant period of mafic fissure eruptions contemporaneous with the development of volcano-tectonic segments along the rift axis. The individual lake basins that comprise the Ziway–Shala system formed asynchronously, and the approximate order of progression is presently constrained by sediment thicknesses and estimated accumulation rates^6^. The first basin to form was the Abijata Main Basin, which has a minimum age of ∼570–330 ka. Between ∼310 and 200 ka faulting initiated along the eastern flank of the CMER^6^, creating the Gedemsa–East Ziway segment (Fig. 1b), and resulted in the initiation of the Ziway Basin. Caldera collapse at the Shala volcanic complex created a deep water reservoir at 240±30 ka (ref. 5) and finally, between ∼200 and 100 ka, tectonic activity in the Abjita basin migrated eastwards and led to the formation of the Langano Basin^6^.

Figure 6 demonstrates that tectonic, volcanic and climatic processes not only operate on comparable timescales but are also coupled. For example, faulting and strain localization controls magma ascent and ponding in the continental crust, which establishes silicic magma reservoirs; tectonic faulting and caldera collapse generate basins, which develop large deep lakes in periods of humidity; lakes and river basins along the rift zone dictate site selection and resource availability for hominin populations; and the establishment of deep groundwater circulation may in turn affect the style of volcanism. To fully understand the links between environmental changes and human evolution, volcanic and tectonic processes^50^,^51^ must be appreciated alongside the palaeoclimate evidence.

The major explosive volcanic eruptions discussed here (DRE volumes of 10–150 km^3^) would have caused rapid and profound environmental disruption in the region. Close to their source vents ignimbrites reach 100–300 m thickness, and mapping^5^ suggests that major collapse ignimbrites had run out distances of 15–40 km. Maps illustrating the scale of tephra fallout for a conservative eruption volume of 5 km^3^ of silicic magma are shown in Fig. 1b. Tephra fall deposits at least 50 cm thick would extend to distances of at least 30 km from the vent, whereas finer fractions >10 cm would be dispersed to distances of ∼100 km. A key implication is that tephra would be dispersed over distances greater than both the width of the rift and the lake basin; potentially closing down along-rift migration corridors. Ash, acidic gases and aerosol released from such events would have affected rift lakes and vegetation causing a cascade of environmental disruption, remodelling the landscapes and resources on which hominins depended. Future tephrochronology studies are required to identify new distal records and evaluate tephra preservation at other archaeological sites along rift, while high-resolution archeological reconstructions (for example, ref. 52) will be critical to evaluating the response of the human landscape to these major geological events (in terms of population declines and relocations).

Pulses of explosive volcanism may typify the East African Rift; each of these would cause significant environmental change, and should not be overlooked as drivers of the population bottlenecks seen in hominin records in East African^11^, as well as a potential ‘push' factor for human dispersal within and out of Africa, along with climate ameliorations in the Late Pleistocene^53^. Geological processes in East African (that is, volcanism and tectonics) occur at rates comparable to Quaternary climatic change (Fig. 6), and the magnitude, timings and feedbacks of these processes must be resolved before we can draw firm links between environmental change and human evolution.

Finally, our hypothesis suggests that the major silicic volcanoes of the MER are currently in a post-caldera stage of evolution (Fig. 6). This would suggest that the likelihood of large caldera-forming eruptions from these volcanoes in the near future is low. Our new eruptive histories (Fig. 3) reveal that small explosive and effusive eruptions have occurred frequently over the last 20 ka, and we emphasize that all of the volcanoes pose a continuing hazard to the region.

## Methods

### ^40^Ar/^39^Ar geochronology

Unweathered rock samples were collected from sites shown on Fig. 2 (coloured stars). Care was taken to avoid sampling lithic fragments in flow units. Rocks were pulverised in a jaw-crusher, and sanidine phenocrysts (250–500 μm in diameter) were separated from the crush using magnetic and lithium metatungstate heavy liquid density separation techniques. Sanidine phenocrysts were leached ultrasonically in 5% hydrofluoric acid for ∼3 min to remove adhering glass and groundmass. Grains were thoroughly rinsed in distilled water and dried, followed by handpicking under a binocular microscope to remove any remaining contaminant phases (fluid/melt inclusions or quartz crystals).

For the Aluto samples sanidine separates were loaded into aluminium disks and irradiated for 30 min in the Cd-lined facility at the TRIGA Reactor, Oregon State University. Grains of the neutron-flux standard Alder Creek sanidine (1.2056±0.0019 Ma, ref. 54) were co-irradiated in the same aluminium disks, in separate wells. Following an ∼2-month cooling period, the Aluto samples and Alder Creek sanidine crystals were analysed at the NERC Argon Isotope Facility, Scottish Universities Environmental Research Centre (SUERC) in a MAP-215 mass spectrometer, with a measured sensitivity of 1.13 × 10^−13^ mol V^−1^. All aliquots were totally fused using a focussed CO_2_ laser, and the released gas purified using two SAES GP50 getters with ST101 Zr-Al cartridges (one at room temperature and the other at 450 °C). Single crystals were analysed for Alder Creek sanidine, and 10–30 single crystals were analysed for each of the Aluto samples to screen for contaminant phases (Supplementary Data 1). All of the single-crystal Aluto analyses yielded single populations of crystals, indicating that no older contaminant phases were identified (Supplementary Data 1). Therefore, to increase the amount of gas analysed for these young samples and improve precision of the analyses, 10 crystals were fused together in each aliquot (Supplementary Data 1) and these multi-crystal analyses were used to determine the ages of the Aluto samples (Supplementary Fig. 1).

Isotope extraction, purification, extraction line operation and mass spectrometry were fully automated. Backgrounds were measured after every two analyses of unknowns. Average backgrounds (±one s.d.) from *n*=133 blank analyses are as follows: ^40^Ar (0.007448±0.0001573 V); ^39^Ar (0.000045±0.000014 V); ^38^Ar (0.000042±0.000029 V); ^37^Ar (0.000457±0.000032 V); and ^36^Ar (0.000099±0.000015 V). Mass discrimination was monitored by analysis of air pipettes after every five analyses. The discrimination factor (*D*=1.0066±0.0024) was calculated using a power law^55^ from the average ^40^Ar/^36^Ar±s.d. (290.8±2.8, *n*=71) of the air pipette data. Analyses of the Alder Creek sanidine samples yielded a *J* factor of 0.0001427±0.000000562. Data were corrected for mass discrimination, nucleogenic interferences and atmospheric contamination using MassSpec software (version 8.058). Values used in the data regression are provided in Supplementary Table 4, while reactor production ratios and interfering isotope production ratios are provided in Supplementary Table 5.

For the Corbetti samples sanidine crystal separates were packaged in Al foil and placed in a cylindrical quartz vial, together with fluence monitors of known age and K-glass and fluorite to measure interfering isotopes from K and Ca. The quartz vials were wrapped in 0.5 mm-thick Cd foil to shield samples from thermal neutrons during irradiation. The samples were irradiated for 1 h in the central thimble of the U.S. Geological Survey TRIGA reactor in Denver, Colorado. The reactor vessel was rotated continuously during irradiation to avoid lateral neutron flux gradients and oscillated vertically to minimize vertical gradients. Reactor constants determined for these irradiations were indistinguishable from recent irradiations, and a weighted mean of constants obtained over the past five years yields ^40^Ar/^39^Ar_K_=0.0010±0.0004, ^39^Ar/^37^Ar_Ca_=0.00071±0.00005 and ^36^Ar/^37^Ar_Ca_=0.000281±0.000006. TCR-2 sanidine from the Taylor Creek Rhyolite^56^ was used as a fluence monitor with an age of 27.87 Ma. Results were recalculated to Fish Canyon sanidine (28.294 Ma, ref. 54) using *R*_TCs/FCs_=1.00881±0.00046, determined in Menlo Park. TCR-2 is a secondary standard calibrated against the primary intralaboratory standard, SB-3, that has an age of 162.9±0.9 Ma (ref. 57). Fluence monitors and unknowns were analysed using a continuous CO_2_ laser system and mass spectrometer described by ref. 58. Gas was purified continuously during extraction using two SAES ST-172 getters operated at 4 and 0 A.

^40^Ar/^39^Ar geochronology was performed at USGS Menlo Park. Discrimination was monitored by analysing splits of atmospheric Ar from a reservoir attached to the extraction line and for these samples *D*_1amu_=1.010795±0.000169. Typical system blanks including mass spectrometer backgrounds were 1.5 × 10^−18^ mol of *m*/*z* 36, 9 × 10^−17^ mol of *m*/*z* 37, 3 × 10^−18^ mol of *m*/*z* 39 and 1.5 × 10^−16^ mol of *m*/*z* 40, where *m*/*z* is mass/charge ratio. Apparent ages were calculated from single-crystal fusion data using atmospheric ratios and are plotted in Supplementary Fig. 2.

All ^40^Ar/^39^Ar geochronology data for the Aluto and Corbetti samples are provided in the Supplementary Data 1 and 2, respectively. Finally, note that all ^40^Ar/^39^Ar and K/Ar ages referred to in the manuscript were calculated (or recalculated) according to the decay constants of ref. 54 and an atmospheric ^40^Ar/^36^Ar ratio of 298.56 (ref. 59). Recalculation of the K/Ar ages for Gedemsa^24^ and Shala^5^ yielded only negligible changes (that is, smaller than the analytical errors) so we quote the ages as originally published.

### Radiocarbon geochronology

Charcoal beneath pumiceous pyroclastic deposits from Aluto was sampled for radiocarbon dating. All pretreatment (acid–alkali–acid) and analytical steps were performed at Beta Analytic Radiocarbon Dating Laboratory (Miami, Florida, USA) and are described in detail at http://www.radiocarbon.com. All radiocarbon dates were calibrated with IntCal13 (ref. 60) and OxCal v.4.2, and the new Aluto radiocarbon results are shown in Supplementary Fig. 3.

### Previous chronologies for Aluto and Corbetti

There are few existing constraints on the ages of eruptive products of Aluto and Corbetti. At Aluto, existing ages range from ∼155 (K/Ar method on a silicic ignimbrite^61^) to 2 ka (radiocarbon age for a post-caldera obsidian flow^62^), while at Corbetti a post-caldera obsidian lava flow on Chabbi yielded a K/Ar age of 20±10 ka (ref. 26). A major complication of the previous geochronological data is that few authors provided precise details of the sampling localities (both geographically and within the context of the stratigraphy) or even details of the material (for example, glass, mineral or organic matter) that was dated, thus limiting the usefulness of the previous chronology and adding considerable uncertainty about the veracity of the ages. This is the case for the age of 155±8 ka for an ignimbrite deposit that was correlated with the caldera forming ignimbrites from Aluto by ref. 61. No details were found regarding the analytical procedure, the precision of the measurement or the sampling locality and we have not considered it within this study. It is important to recognize that re-analysis of K/Ar chronologies using ^40^Ar/^39^Ar often leads to considerable age revisions (for example, ref. 12).

### Volume calculations

To obtain a first-order approximation of the volume of silicic magma produced at each volcano through time we consider their eruptive histories to be comprise three main phases: a pre-collapse edifice; caldera collapse; and post-caldera volcanism (Fig. 4). These stages are a reasonable approximation of the long-term eruptive cycle of silicic peralkaline volcanoes^25^ and also agree with the existing constraints on the eruptive histories of the volcanic complexes considered in this study^5^,^16^,^17^,^24^.

For the pre-collapse edifice we characterize the volcano as a simple conical structure; the centre of the pre-collapse volcano is equivalent to the centre of the present-day caldera, and the slope of the pre-collapse volcano is calculated using the mean slope of the caldera walls away from the complex (for example, Supplementary Fig. 4) using a present-day DEM (we used SRTM 1 Arc-Second Global elevation data at a resolution of 30 m, the highest-resolution, freely available topographic data available at this time). We then use thickness estimates of pre-caldera lavas from geological mapping, usually taken from caldera wall sequences, as the final variable to approximate the volume of the pre-collapse cone.

Calderas are volcanic collapse features related to withdrawal of magma from an underlying reservoir^25^,^63^. Since most calderas can be approximated by a subsiding block surrounded by a ring fault^31^,^32^, we assume that the calderas considered in this study consist of a cylindrical piston (caldera block) above a magma reservoir. Thus, the net volume of the caldera can be used to estimate the volume of material ejected by the caldera-forming eruption. Kandlbauer and Sparks^33^ compared different methods for calculating eruptive volumes and found that estimates of caldera volumes compare well with those from ash fall and ignimbrite thickness extrapolations^64^,^65^, supporting the method we have used here. We assume that the cylindrical caldera collapse volume is defined by the long and short axis of the caldera structure and depth is defined by the present-day caldera wall height. Following observations of ref. 32 we assume that intra-caldera ignimbrite deposits partially infill the caldera floor. At Aluto volcano, deep well stratigraphy records confirm thick sequences (100–300 m) of caldera-filling ignimbrites^47^. Deep-well records only exist for Aluto and therefore we have assumed that similar thicknesses of fill ignimbrites also exist at the other caldera complexes. The total volume of the caldera collapse is calculated by summing the present-day volume of the caldera and the volume of the intra-caldera fill (approximated as a cylinder with equivalent spatial dimensions to the caldera structure).

Following caldera collapse each volcano has undergone further (post-caldera) lava extrusion. The volume of the post-caldera deposits was calculated using DEMs following the method of ref. 34. For each volcano, polygons outlining the post-caldera deposits were created and used to mask out these volcanic landforms from the DEM. The removed region of the DEM was then interpolated using a variable tension parameter to derive a smoothed near-flat pre-eruption surface across the masked area. We then subtracted the present-day DEM from the interpolated DEM (for example, Supplementary Fig. 5a minus Supplementary Fig. 5c) and calculate the residual volume between the surfaces, thus providing a volume estimate for the post-caldera eruptive products. DEM interpolations and volume calculations were carried out using GMT software^34^. This method calculates a minimum post-caldera volume, as it only accounts for proximal volcanic deposits rather than distal tephra dispersed far beyond the vent. Our mapping suggests that the bulk of post-caldera deposits are typified by localized pyroclastic flows and coulees (Fig. 2) rather than widely dispersed pumice fall units, and until detailed correlations of the post-caldera tephra sequences are completed the DEM differencing method (above) is the most appropriate method to use.

Eruptive volumes were then converted to a DRE. For the pre- and post-caldera edifice we assume that the deposits comprise a 50:50 mix of silicic lava and volcaniclastic sequences (with bulk densities of 2,200 and 1,800 kg m^−3^, respectively^66^). DRE volumes are calculated assuming a (water-free) silicic magma density of 2,380 kg m^−3^ (ref. 67). The caldera volume is assumed to be equivalent to the volume of magma withdrawn from the reservoir and so no conversion is applied. For each volcanic complex the constraints on eruptive history and assumptions made in volume calculations are outlined in Supplementary Tables 1–3 and accompanied by Supplementary Figs 4–10.

### Geochemical overview and the links between Pliocene and Pleistocene magmas

WoldeGabriel *et al*.^68^ noted a spatial overlap between certain Pliocene calderas (for example, Munesa) and Pleistocene volcanic centres (for example, Shala and Aluto) suggesting that the youngest edifices are built on pre-existing structures (such as ring fractures). At present there is little structural or geophysical evidence to support this and future work to establish whether any links exist is to be encouraged. It might also be speculated that the flare-up could be caused by rejuvenation of residual silicic magmas originally emplaced beneath the Pliocene volcanic centres. Although geochemical data from the CMER are limited, in Supplementary Fig. 11 we compare a selection of whole rock major and trace element data from Aluto^69^ with an ignimbrite collected from Munesa (after ref. 47, dated to ∼2.3 Ma). Incompatible trace element data from pre-, syn- and post-caldera deposits from Aluto show relative continuity and form a positive linear trend is consistent with a single magmatic lineage formed through extensive fractional crystallization. This observation is consistent with geochemical constraints from Gedemsa^24^,^70^,^71^, which suggest that fractional crystallization (plus minor contamination) rather than partial melting processes explain the petrogenetic origins of these peralkaline melts. The Munesa ignimbrite falls off the main linear array evidencing a different fractionation trend and a distinct petrogenetic evolution from the Aluto samples. Therefore we conclude that the available geochemical evidence (Supplementary Fig. 11) does not require a genetic link between the Pliocene and Pleistocene magmas.

### Data availability

The data that support the findings of this study are available are available within the article and its Supplementary Information Files.

## Additional information

**How to cite this article:** Hutchison, W. *et al*. A pulse of mid-Pleistocene rift volcanism in Ethiopia at the dawn of modern humans. *Nat. Commun.* **7,** 13192 doi: 10.1038/ncomms13192 (2016).

## Supplementary Material

Supplementary Information Supplementary Figures 1-11, Supplementary Tables 1-5 and Supplementary References

Supplementary Data 1 ^40^Ar/^39^Ar age data table for Aluto (analysis conducted at SUERC).

Supplementary Data 2 ^40^Ar/^39^Ar age data table for Corbetti (analysis conducted at USGS Menlo Park). a. COI2E - Sanidine. b. Chabbi 7 - Sanidine.

## Figures and Tables

**Table 1:** Age constraints on silicic volcanism at Aluto and Corbetti volcanoes.

**Volcano**	**Locality**	**Sample code(s)**	**Rock type**	**Method**	**Age**	**Additional notes and references**
Aluto	6 km SSE of edifice	18-11-07	Welded ignimbrite (grey)	^40^Ar/^39^Ar (sanidine)	316±19 (316±12) ka	This study
	2 km NW of edifice	13-05-04	Welded ignimbrite (green)	^40^Ar/^39^Ar (sanidine)	306±12 (306±8) ka	This study
	SE flank of edifice	18-02-04	Comenditic rhyolite	^40^Ar/^39^Ar (sanidine)	55±19 (55±9) ka	This study
	N flank of edifice	30-01-LNE	Vitrophyric obsidian	^40^Ar/^39^Ar (sanidine)	19±5 (18±8) ka	This study
	E flank of edifice	31-01-LE	Vitrophyric obsidian	^40^Ar/^39^Ar (sanidine)	16±14 (12±7) ka	This study
	Deka Wede (DW)	Gif-3987, SUA-494	Tephra	^14^C (lacustrine silt and charcoal)	26.41±0.64 to 13.86±0.43 cal. ka BP	Volcaniclastic sequence with ∼13 distinct tephra layers^6^,^15^,^18^
	Deka Wede (DW)	Gif-3986, Gif-3984	Tephra	^14^C (lacustrine marl and charcoal)	12.26±0.27 to 9.55±0.28 cal. ka BP	Pumice overlying fluvial sands, above the Abernosa pumice^6^,^15^,^18^
	Deka Wede (DW)	Gif-3984	Tephra	^14^C (charcoal and melanoides shells)	9.55±0.28 to 6.60±0.13 cal. ka BP	Pumice overlying palaeosol, youngest tephra at Deka Wede^6^,^15^,^18^
	Haroresa (HR)	2a, 2b	Tephra	^14^C (melanoides shells)	11.12±0.11 to 7.29±0.06 cal. ka BP	Pumice bracketed by gravels, youngest tephra at Haroresa^19^
	E flank of edifice	31-01-08	Tephra	^14^C (charcoal)	0.40±0.05 cal. ka BP	This study, youngest pumiceous pyroclastic deposits identified on edifice
Corbetti	WNW caldera wall	COI2E	Welded ignimbrite (red)	^40^Ar/^39^Ar (sanidine)	182±28 (177±4) ka	This study
	E flank of Chabbi	Chabbi-7	Vitrophyric obsidian	^40^Ar/^39^Ar (sanidine)	19±15 (18±4) ka	This study
	4 km E of Chabbi	WKSC14A	Tephra	^14^C (palaeosol)	2.34±0.04 cal. ka BP	Pumice overlying palaeosol, youngest widespread tephra^17^

See Methods section for analytical details. Full experimental results are provided in Supplementary Figs 1–3 and Supplementary Data 1 and 2.

Radiocarbon ages (^14^C) are given as the mean±one s.d. of the calibrated range at the 95.4% probability range.

^40^Ar/^39^Ar ages shown are the arithmetic mean±one s.d., and in brackets the weighted mean±s.e.m. (1*σ*), we quote the former throughout the manuscript.

SSE, south-southeast; NW, northwest; SE, southeast; N, north; E, east; WNW, west-northwest.
