# Supplementary material for: A pulse of mid-Pleistocene rift volcanism in Ethiopia at the dawn of modern humans
Source: Nat Commun. 2016 Oct 18;7:13192. doi: 10.1038/ncomms13192 (PMC5071850; doi:10.1038/ncomms13192)
Supplement: Supplementary Information — Supplementary Figures 1-11, Supplementary Tables 1-5 and Supplementary References [file ncomms13192-s1.pdf]

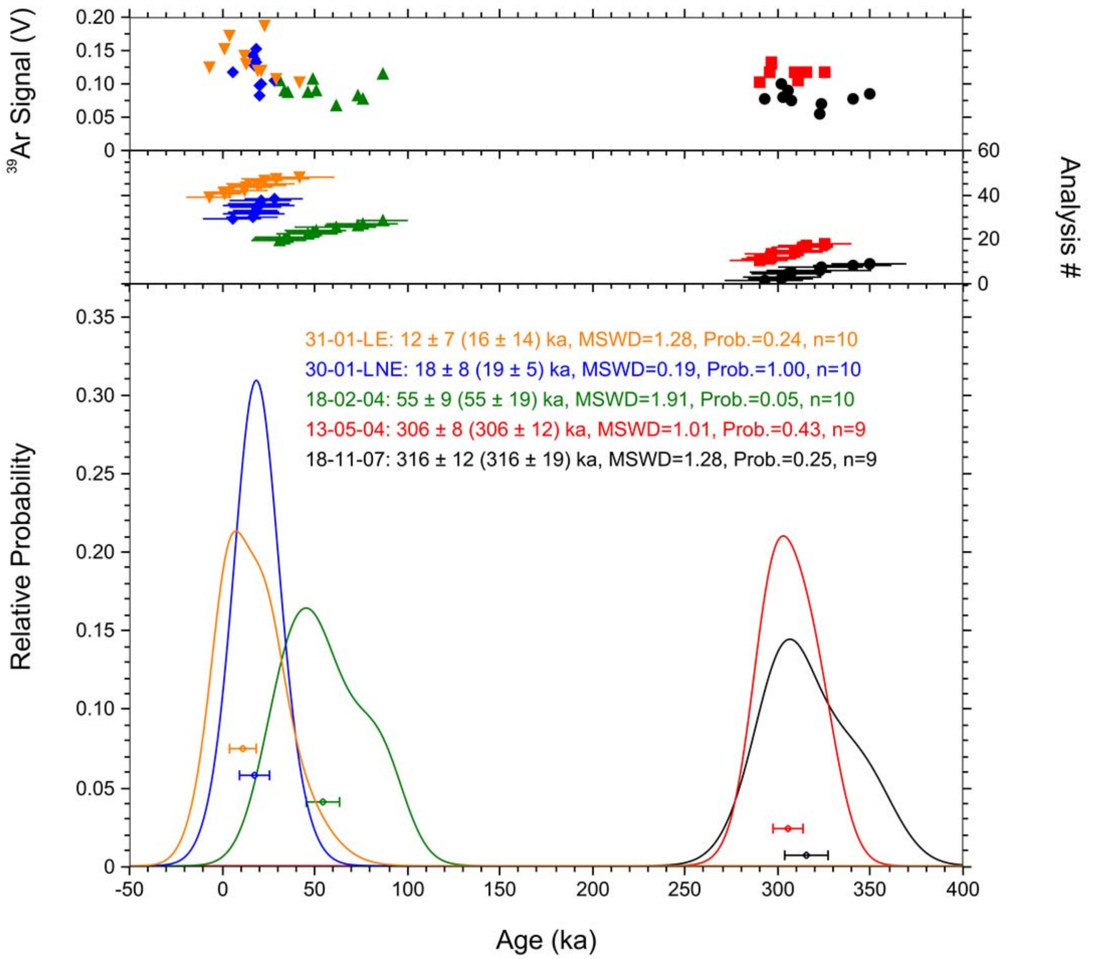

Supplementary Figure 1. Age probability spectra for SUERC $^{40}Ar/^{39}Ar$ sanidine analyses from Aluto. Samples 18-11-07, 13-05-04, 18-02-04, 30-01-LNE and 31-01-LE are shown. Ages shown are the weighted mean ± standard error of the mean (1σ), and in brackets the arithmetic mean ± one standard deviation. While both methods of error reporting are equally valid, for consistency throughout the article, we take precedent from previous hominin literature[1] and report ages as arithmetic mean ± one standard deviation.

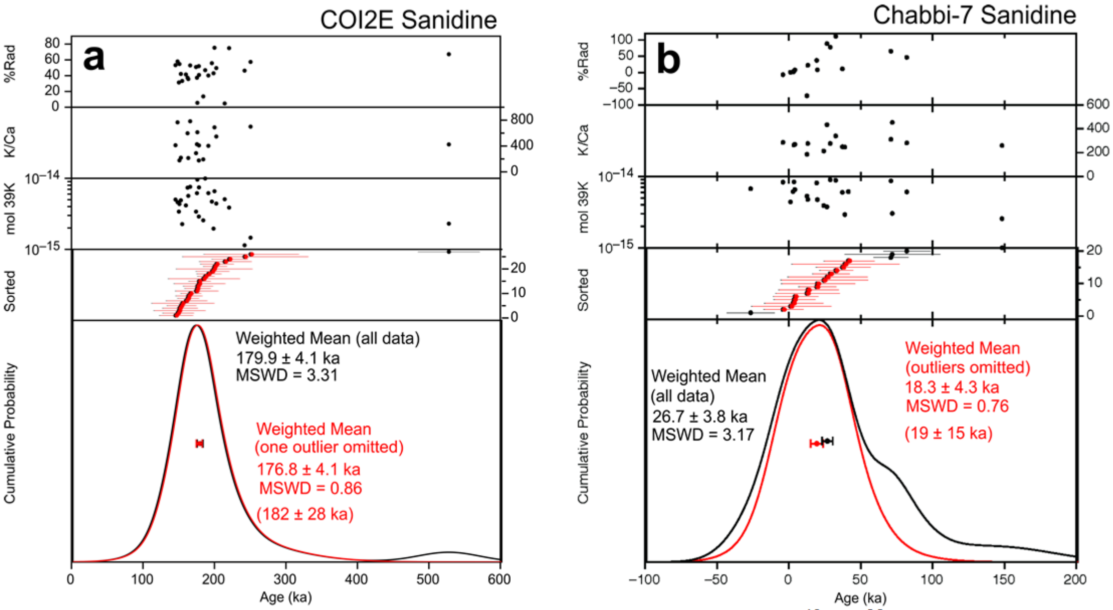

Supplementary Figure 2. Age probability spectrum for USGS $^{40}Ar/^{39}Ar$ sanidine analyses from Corbetti. a. Sample COI2E and **b.** Sample Chabbi-7. Ages shown are the weighted mean ± standard error of the mean (1σ), and in brackets the arithmetic mean ± one standard deviation.

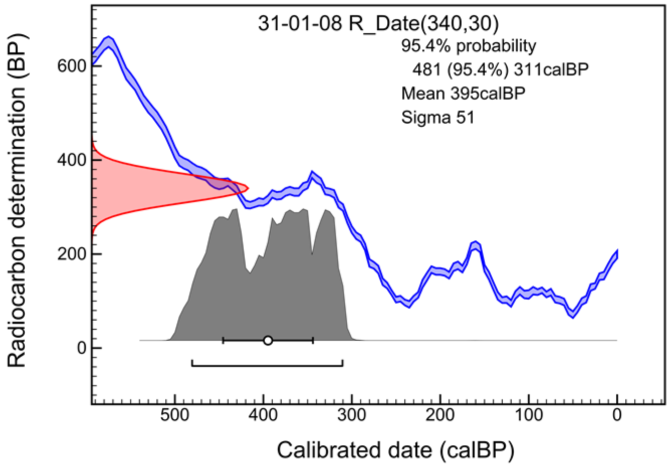

Supplementary Figure 3. Calibration of the radiocarbon age for the sample 31-01-08. The charcoal sample was collected beneath young pumiceous pyroclastic deposits on the east of the Aluto edifice. Radiocarbon dates were calibrated with IntCal13[2] and OxCal v.4.2.

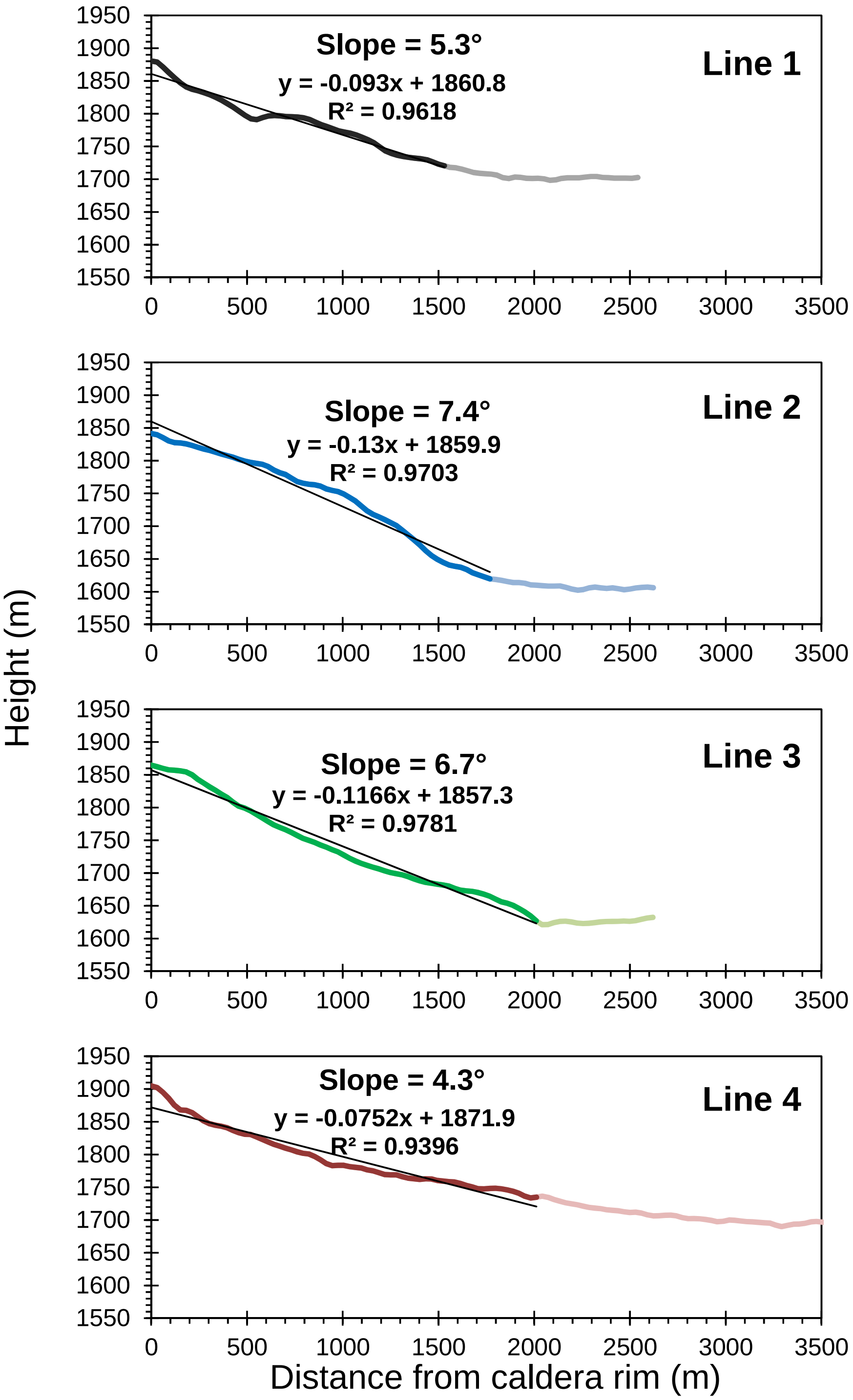

Supplementary Figure 4. Topographic profiles constructed away from the caldera walls of Gedemsa volcano. Profile are used to estimate the slope of the pre-collapse volcano, and combined with thickness estimates to calculate the volume of the pre-collapse edifice, see Methods section for details. For each profile the slope measurement (i.e., the darker coloured segment of line) was made between the caldera rim and the point at which flat lying rift floor sediments were intersected (i.e., light coloured segment of line). Profile lines are shown in map view in Supplementary Figure 5.

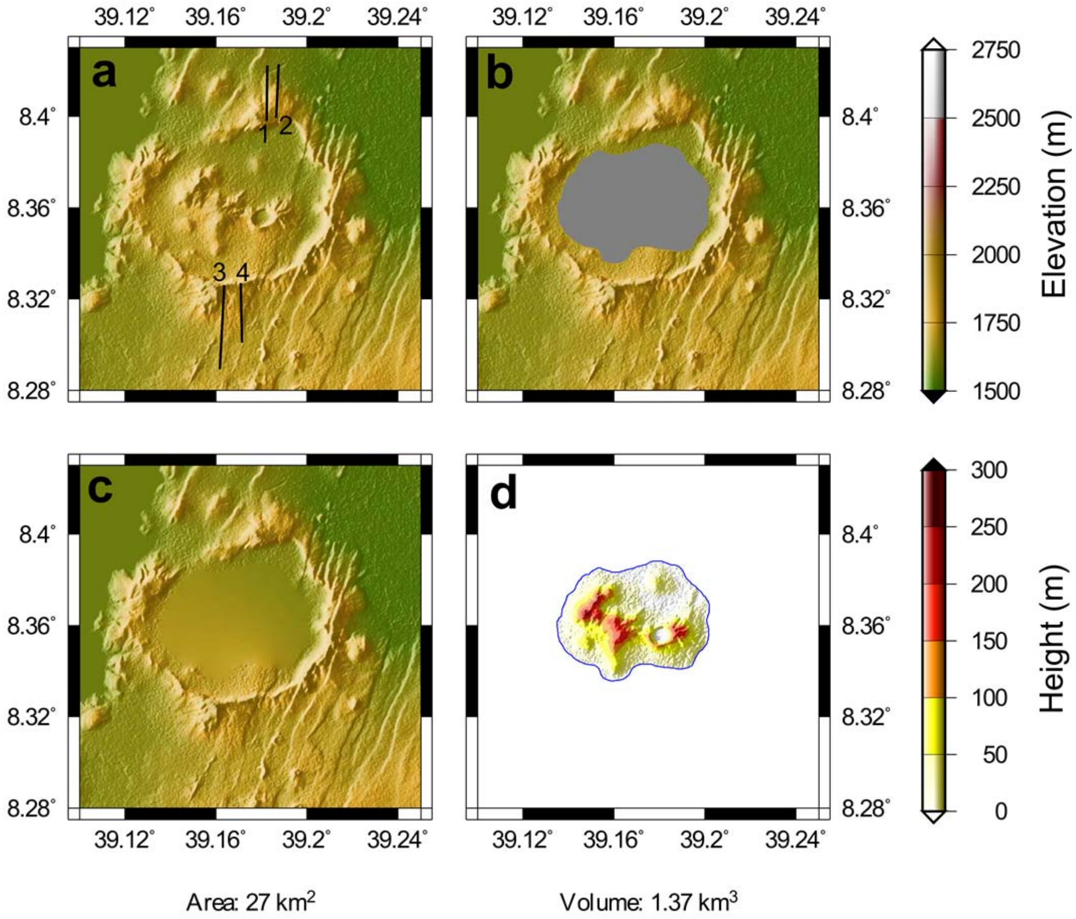

Supplementary Figure 5. DEM differencing method used to mask out post-caldera deposits at Gedemsa and calculate their volume. a. represents the present-day DEM, and lines 1–4 correspond to the topographic profiles given in Supplementary Figure 4. **b.** shows the masked area (grey). **c.** shows the present-day DEM with interpolated masked surface and **d.** shows the difference between a and c, shown as residual height. The area of the masked region is given in the bottom left, while the volume of masked region (i.e., the volume of post-caldera deposits) is given in the bottom right. Note that volume shown is not corrected to a dense rock equivalent (DRE).

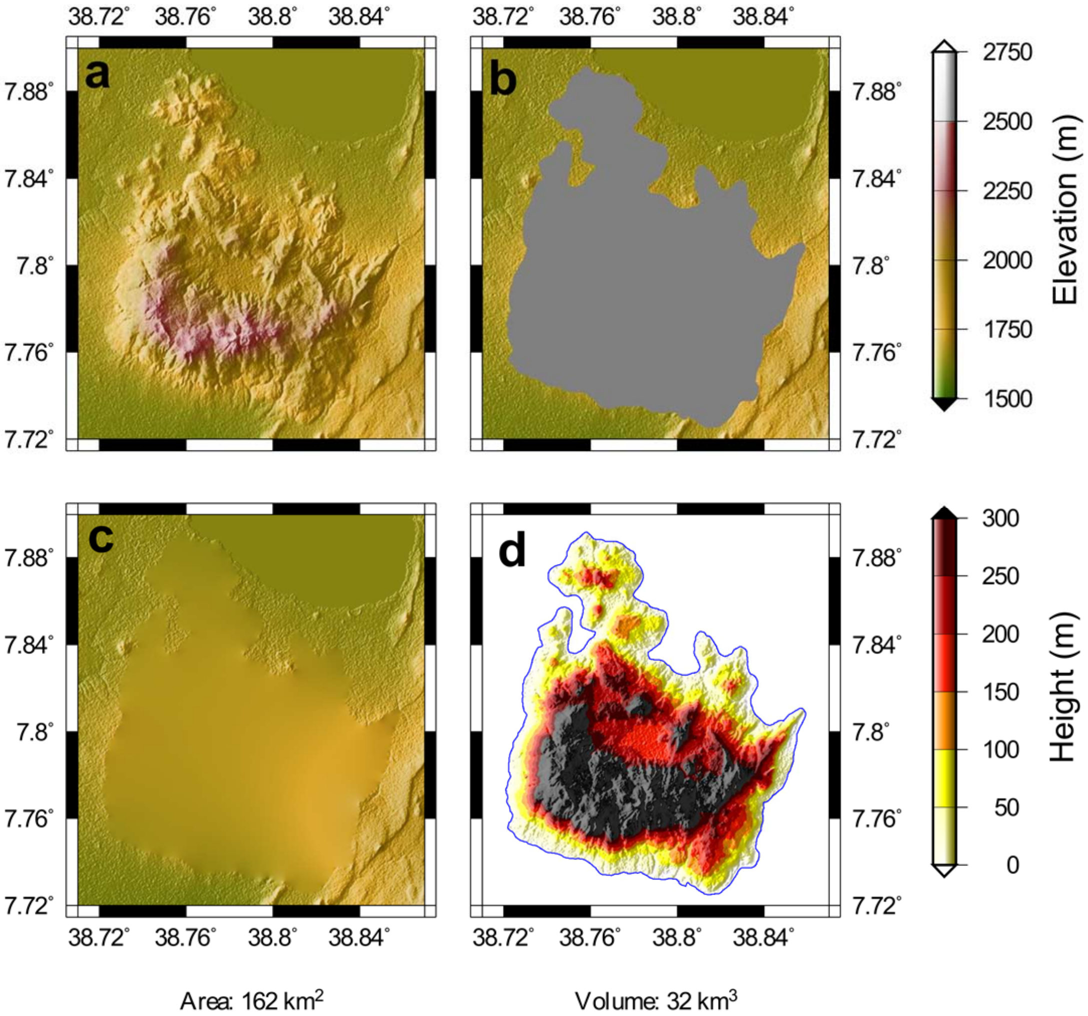

Supplementary Figure 6. DEM differencing method used to mask out and calculate the volume of the main edifice of Aluto. We approximate this as the volume of all post-caldera deposits (see Supplementary Table 3 for further details). **a.** represents the present-day DEM. **b.** shows the masked area (grey). **c.** shows the present-day DEM with interpolated masked surface and **d.** shows the difference between a and c, shown as residual height. The area of the masked region is given in the bottom left, while the volume of masked region (i.e., the volume of edifice) is given in the bottom right. Note that volume shown is not corrected to a dense rock equivalent (DRE).

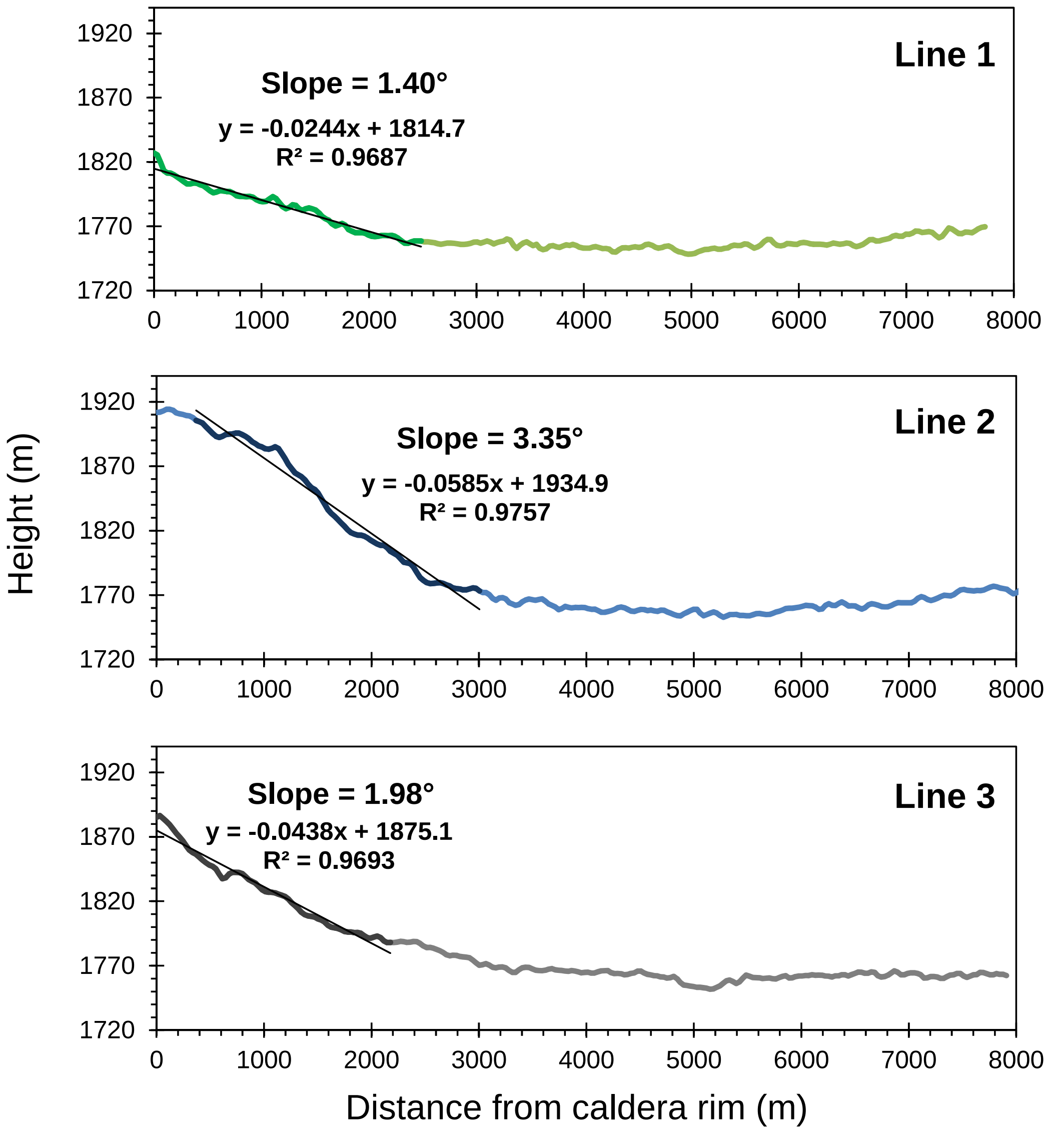

Supplementary Figure 7. Topographic profiles constructed away from the caldera walls of Shala volcano. Profile are used to estimate the slope of the pre-collapse volcano, and combined with thickness estimates to calculate the volume of the pre-collapse edifice, see Methods section for details). For each profile the slope measurement (i.e., the darker coloured segment of line) was made between the caldera rim and the point at which flat lying rift floor sediments were intersected (i.e., light coloured segment of line). Profile lines are shown in map view in Supplementary Figure 8.

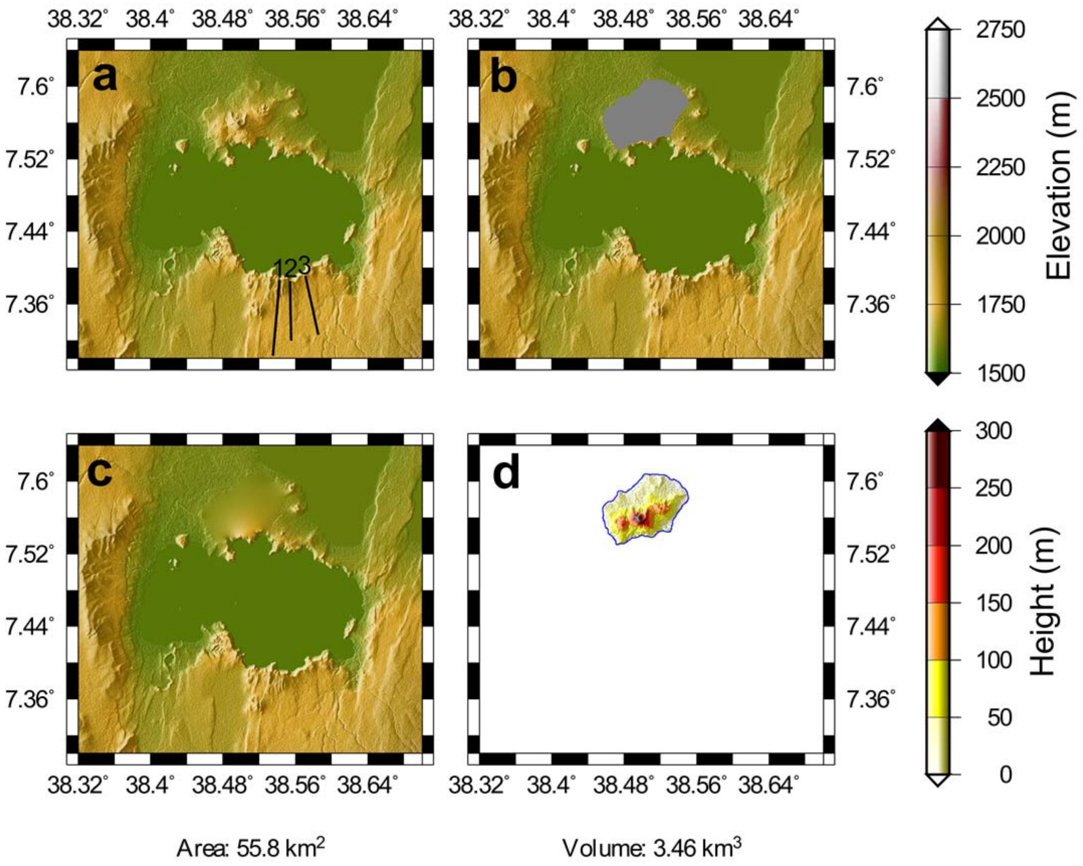

Supplementary Figure 8. DEM differencing method used to mask out post-caldera deposits at Shala and calculate their volume. **a.** represents the present-day DEM, and lines 1–3 correspond to the topographic profiles given in Supplementary Figure 7. **b.** shows the masked area (grey). **c.** shows the present-day DEM with interpolated masked surface and **d.** shows the difference between a and c, shown as residual height. The area of the masked region is given in the bottom left, while the volume of masked region (i.e., the volume of post-caldera deposits) is given in the bottom right. Note that volume shown is not corrected to a dense rock equivalent (DRE).

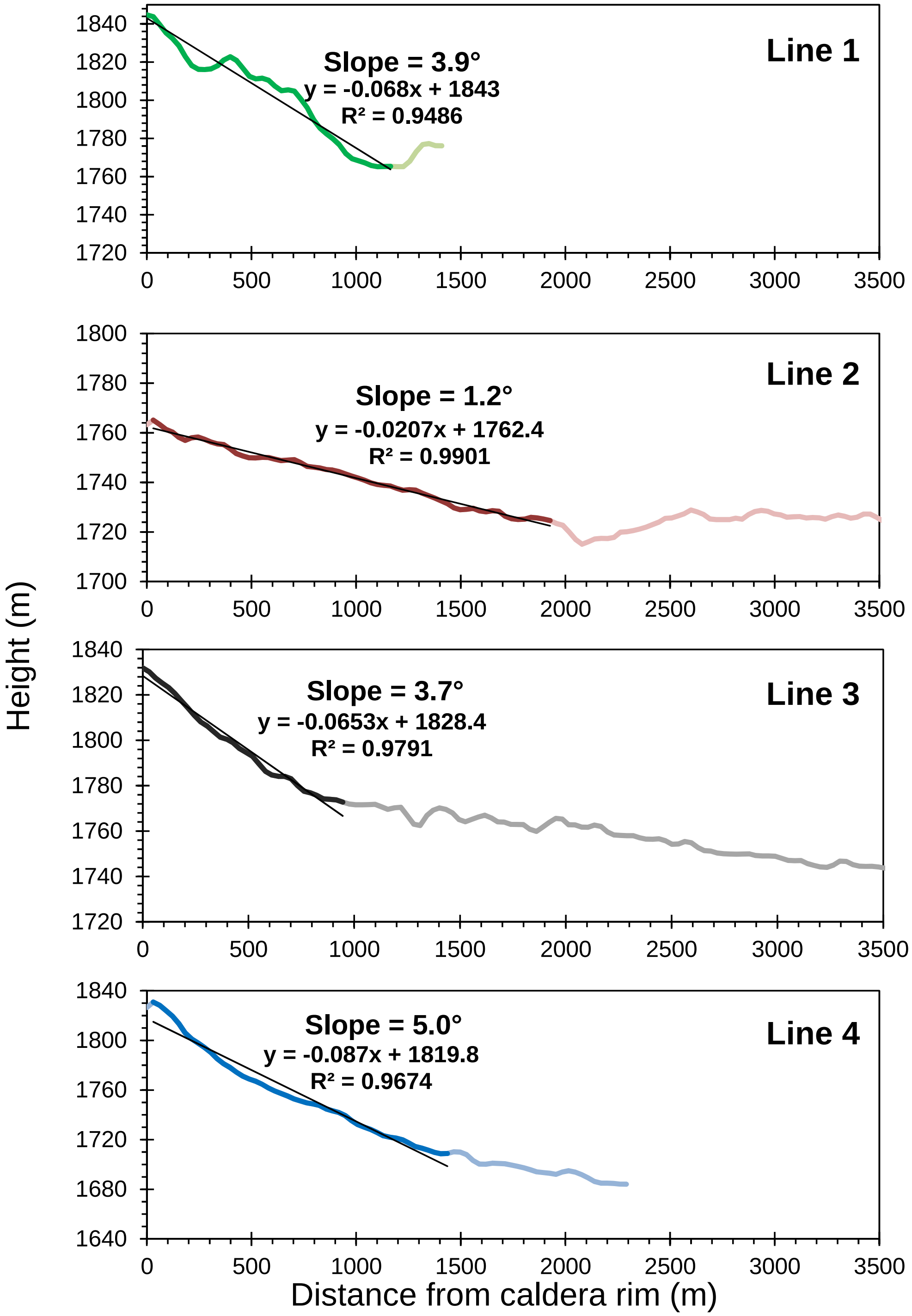

Supplementary Figure 9. Topographic profiles constructed away from the caldera walls of Corbetti volcano. Profiles are used to estimate the slope of the pre-collapse volcano, and combined with thickness estimates to calculate the volume of the pre-collapse edifice, see Methods section for details. For each profile the slope measurement (i.e., the darker coloured segment of line) was made between the caldera rim and the point at which flat lying rift floor sediments were intersected (i.e., light coloured segment of line). Profile lines are shown in map view in Supplementary Figure 10.

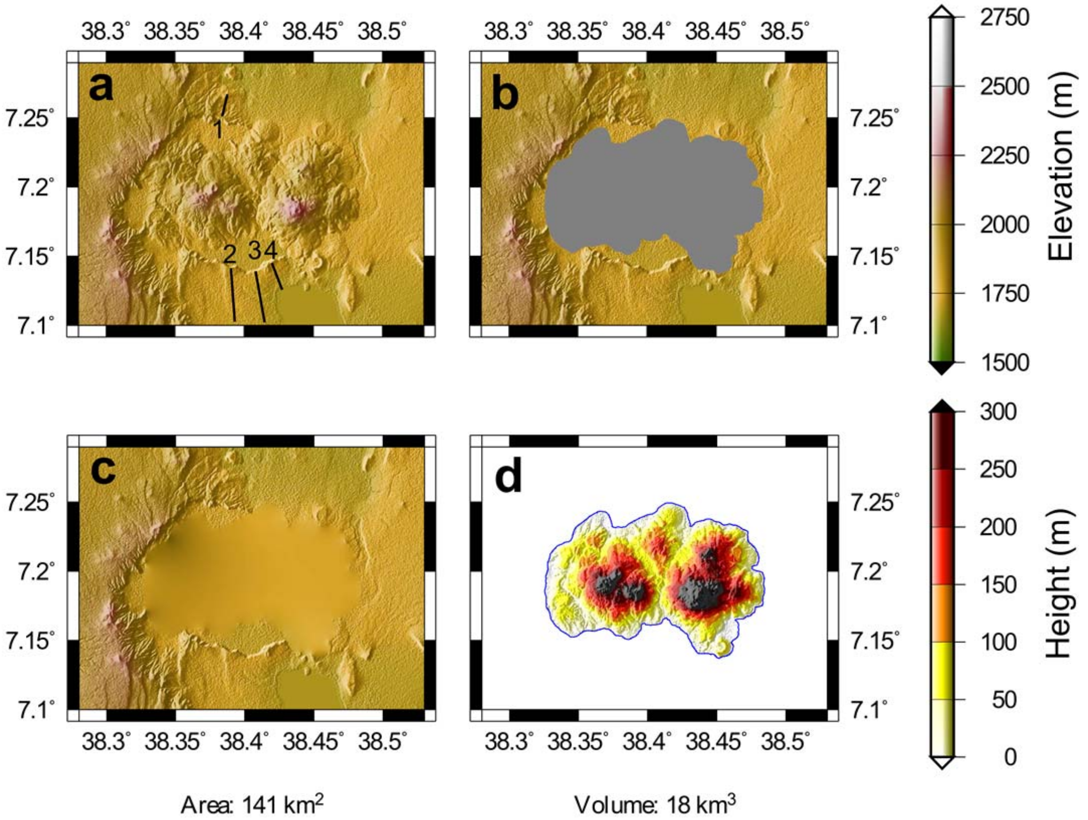

Supplementary Figure 10. DEM differencing method used to mask out post-caldera deposits at Corbetti and calculate their volume. a. represents the present-day DEM, and lines 1–4 correspond to the topographic profiles given in Supplementary Figure 9. **b.** shows the masked area (grey). **c.** shows the present-day DEM with interpolated masked surface and **d.** shows the difference between a and c, shown as residual height. The area of the masked region is given in the bottom left, while the volume of masked region (i.e., the volume of post-caldera deposits) is given in the bottom right. Note that volume shown is not corrected to a dense rock equivalent (DRE).

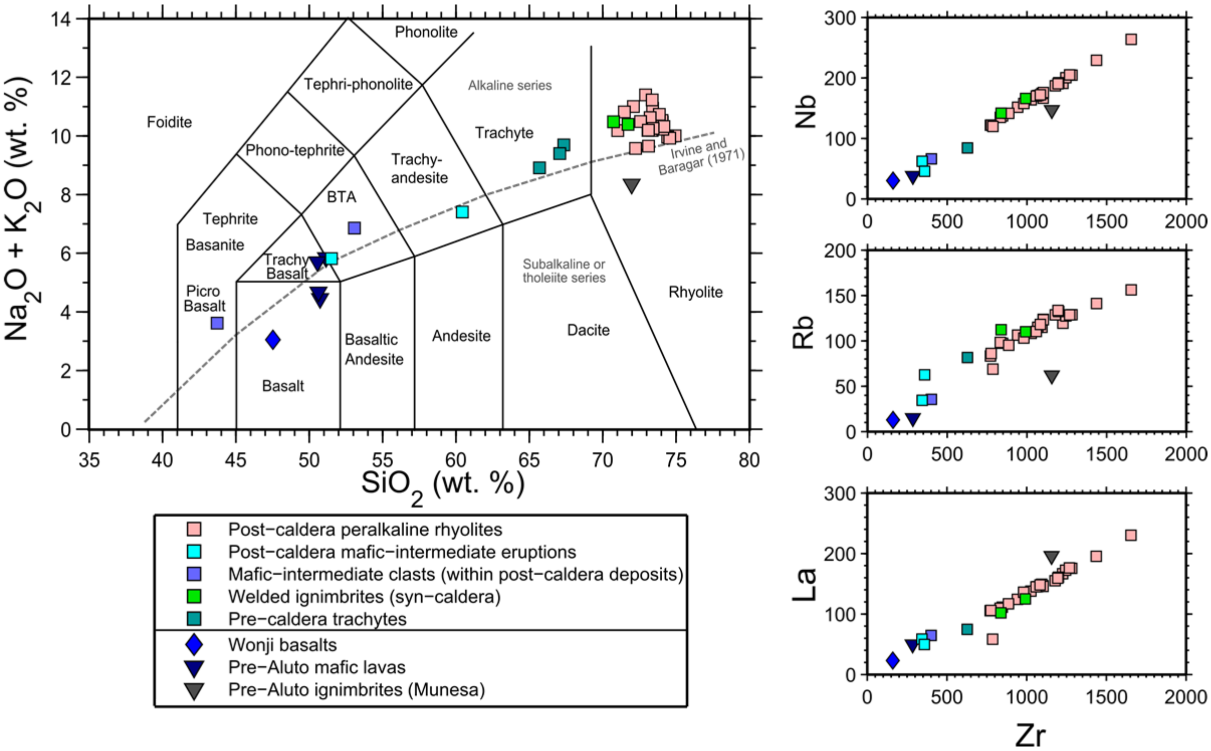

Supplementary Figure 11. Overview of whole-rock geochemical data from Aluto volcano and the surrounding region. Major elements were determined by XRF and are shown in wt. % while trace elements were determined by both XRF and ICP-MS methods and are shown in ppm (data after refs. 3,4). The upper left plot shows a total alkalis versus silica (TAS) diagram[5]. The grey dashed line shows the alkaline-sub-alkaline divide of ref. 6. The right hand plots show incompatible elements (Nb, Rb and La) plotted against Zr. While the Munesa ignimbrite (grey inverted triangle, analysed by ref. 3) is broadly comparable to the Aluto rhyolites in major element chemistry, incompatible element plots demonstrate that the Munesa sample falls off the main linear array and evidences a different fractionation trend from the Aluto samples. We conclude that the available geochemical evidence does not require a genetic link between the Munesa (Pliocene) and pre-, syn- and post- caldera (Pleistocene) magmas from Aluto.

Supplementary Table 1. Pre-caldera edifice volume estimates.

	Estimate	Caldera wall thicknesses (m)	Uplifted fault section thickness (m)*	Edifice slope (°)	Volume DRE (km^3)	Further details on volume estimate
**Gedemsa**	High	300	-	7.5	35	The pre-caldera volcanic sequences (Fig. 2d) are dominated by rhyolitic lavas, although pumice fall and ignimbrite deposits are also present[7,8]. Individual rhyolite lava flows are thick (20–30 m), 2–5 km long and show a clear flow folding[8]. Based on the geological map of ref. 7 the minimum thickness of the pre-caldera deposits, identified in the northern caldera wall of Gedemsa, is 250 m. Slope measurements, made on the northern and southern back walls of the caldera (i.e., the non-faulted sections) range between 4–7.5° (Supplementary Figure 4).
	Med	250	-	6	28	
	Low	150	-	4	18	
**Aluto**	High	-	200	5	51	Evidence for a pre-collapse edifice is observed along faults on the margins of the complex (Figs. 2a, 3) where a minimum thickness (~100 m) of trachyte lavas and tuffs ~8 km from the centre of the present edifice are found. Our interpretation is that trachyte lava flows and tuffs built up a low relief silicic complex (or lava shield) upon the faulted rift terrain. The pre-caldera edifice has been largely covered by recent volcanic deposits, and so we assume that the slope of the pre-caldera edifice is in line with that of neighbouring complexes (3–5°).
	Med	-	150	4	40	
	Low	-	100	3	29	
**Shala**	High	200	-	5	51	Mohr and colleagues (ref. 9) consider the pre-caldera edifice to have comprised a thick pile of flows and domes of rhyolite. Sites sampled at the caldera wall show well preserved sequences and a minimum thicknesses of trachyte and rhyolite for the pre-collapse edifice is 50 –150 m (ref. 9). Measurements of slope on the southern caldera wall (N.B. other wall sections are deeply eroded and/or faulted) show values of 2–4° (Supplementary Figure 7).
	Med	100	-	4	37	
	Low	50	-	3	26	
**Corbetti**	High	200	-	5	35	Silicic lava domes and flows are reported in caldera wall sequences of Corbetti and pre-date the caldera-forming eruption[10,11] (Fig. 2b). The pre-caldera lava exposures are well in excess of 50 m, and we consider a range of 100–200 m as a reasonable approximation of their true thickness. The range of slopes for the Corbetti caldera walls are shown in Supplementary Figure 9.
	Med	150	-	3.5	25	
	Low	100	-	1.5	12	

* At Aluto recent (post-caldera volcanism) has obscured much of the original caldera wall and so we use fault exposures off the main edifice to evaluate pre-caldera thicknesses

Supplementary Table 2. Caldera collapse volume estimates.

	Estimate	Caldera wall height (m)	Intra-caldera fill (m)	Volume DRE (km^3)	Further details on volume estimate
**Gedemsa**	High	250	300	31	Outline mapping by refs. 7,8 identified a pyroclastic sequence of breccias and welded ignimbrite deposits overlying the pre-caldera sequences. It has been suggested[7,8] that caldera collapse may not simply relate to a single event and that multiple explosive eruptions, occurring in different sectors of the volcano, over a 320–260 ka period generated the caldera. However, until detailed mapping of these individual eruptions takes place it is reasonable to assume that the bulk caldera subsidence over the collapse period is represented by the present-day (8 km × 9 km) caldera structure which has typical wall heights between 150–250 m.
	Med	200	200	23	
	Low	150	100	14	
**Aluto**	High	200	300	21	The ring structure is hypothesized to be 9 km × 6 km and with a minimum height of ~100 m (ref. 12). Deep well stratigraphy records[13,14] reveal grey-green welded ignimbrites, variably consolidated tuffs and lithic breccias overlying lacustrine sediments and pre-Aluto mafic lava sequences. The deep well ignimbrites also underlie the obsidian lava flows that characterize the post-caldera deposits. The deep well ignimbrites have a fine, devitrified groundmass, and contain sanidine phenocrysts as well as rhyolitic and trachytic lithic fragments. We correlate these units with the surface deposits of welded grey and green ignimbrite sequences (Fig. 2) that yielded overlapping ^{40}Ar/^{39}Ar ages of 316 ± 13 ka and 306 ± 12 ka (Fig. 2). The deep well ignimbrite sequences are thickest in the centre of the complex where they are in excess of 300 m in well LA-8[14].
	Med	150	200	15	
	Low	100	100	8	
**Shala**	High	610	300	170	Collapse at Shala is represented by the submerged caldera, 18 km east-west and 14 km north-south (Supplementary Figure 8). The maximum depth of Lake Shala, 260 m (ref. 15), is added to the height of the caldera wall above the lake surface (ranging from 100–350 m). The erupted volume of 86–170 km^3 estimated for the Shala caldera agrees well with previous estimates of 120 km^3 reported by refs. 9,16.
	Med	510	200	133	
	Low	360	100	86	
**Corbetti**	High	200	300	63	The Corbetti caldera (14 km × 11 km) has wall heights of 100–200 m and on the outskirts of the caldera collapse deposits reach 200 m thickness (ref. 10).
	Med	150	200	44	
	Low	100	100	25	

Supplementary Table 3. Post-caldera volume estimates.

	Area (km^2)	Volume DRE (km^3)	Further details on volume estimate
**Gedemsa**	27	1	Post-caldera volcanism has generated a set of small coalescing edifices (200–250 m high) in the centre of the caldera (Fig. 2d). The post-caldera eruptive units are composed of alternating rhyolitic lavas and pumice-fallout deposits
**Aluto**	162	27	Several phases of post-caldera silicic volcanism have occurred at Aluto over the last ~60 ka (Fig. 3), and deep wells sited in the centre of the complex reveal that post-caldera units (peralkaline rhyolite lavas and pyroclastic units) are ~250 m in thickness[3,17]. Estimating the volume of these units using the DEM masking approach is complicated because the post-caldera deposits do not have a clearly defined morphology (i.e., they mantle the pre-existing volcanic collapse feature, rather than fill the caldera floor). The total volume of the present edifice is 32 km^3 (Supplementary Figure 6), this represents a relatively high estimate of the total volume of post-caldera products because the collapse topography, buried beneath the surface, may be included as part of the volume. A simplified approach to calculate the total volume of the post-caldera units at Aluto, is to assume they have a constant thickness (250 m, after deep well observations[3,17]) proximal to the volcanic centre, equivalent to a cylinder of 6 km radius, and this yields a total volume of 28 km^3. This value is almost identical to the total volume of the current edifice (Supplementary Figure 6), and while the good agreement is probably partly fortuitous, it indicates that an estimate of ~30 km^3 is the correct order of magnitude and therefore, following conversion to DRE, we take the volume of post-caldera collapse products at Aluto as 27 km^3.
**Shala**	56	3	Post-caldera volcanism has been limited to the Tulu Fike pyroclastic cone on the northern shore of Shala lake[9] (Fig. 2c).
**Corbetti**	141	15	Post-caldera activity at Corbetti is represented by two volcanic cones (Urji and Chabbi, Fig. 2b) formed of pumice flows, pumice fall and obsidian coulees[18].

Supplementary Table 4. Values used in the data regression.

Term	Value used	Reference
$(^{40}Ar/^{36}Ar)_{Atm}$	298.56 ± 0.31	(ref. 19)
$^{40}K/K$	1.167×10^{-4} mol/mol	(ref. 20)
^{40}K $\lambda\varepsilon$	$(5.757 \pm 0.016) \times 10^{-11}$ a^{-1}	(ref. 21)
^{40}K λ_{β}	$(4.9548 \pm 0.0134) \times 10^{-10}$ a^{-1}	(ref. 21)
^{40}K λ total	$(5.5305 \pm 0.0135) \times 10^{-10}$ a^{-1}	(ref. 21)
^{37}Ar λ	0.01983 ± .0000226 $days^{-1}$	(ref. 22)
^{39}Ar λ	$(7.055 \pm 0.039) \times 10^{-6}$ $days^{-1}$	(ref. 23)
^{36}Cl λ_{β}	$(6.1817 \pm 0.040) \times 10^{-9}$ $days^{-1}$	(ref. 24)

Supplementary Table 5. Reactor production ratios and interfering isotope production ratios.

Term	Value used	Reference
$(^{36}Cl/^{38}Cl)_{Cl}$	263 ± 2	(ref. 24)
$(^{38}Ar/^{37}Ar)_{Ca}$	$(1.96 \pm 0.08) \times 10^{-5}$	(ref. 25)
$(^{38}Ar/^{39}Ar)_{K}$	$(1.22 \pm 0.01) \times 10^{-2}$	(ref. 25)
$(^{40}Ar/^{39}Ar)_{K}$	$(7.30 \pm 0.92) \times 10^{-4}$	(ref. 25)
$(^{37}Ar/^{39}Ar)_{K}$	$(2.24 \pm 0.16) \times 10^{-4}$	(ref. 25)
$(^{39}Ar/^{37}Ar)_{Ca}$	$(6.95 \pm 0.09) \times 10^{-4}$	(ref. 25)
$(^{36}Ar/^{37}Ar)_{Ca}$	$(2.65 \pm 0.02) \times 10^{-4}$	(ref. 25)

Supplementary references

1. McDougall, I., Brown, F. H. & Fleagle, J. G. Stratigraphic placement and age of modern humans from Kibish, Ethiopia. *Nature* **433,** 733–736 (2005).
2. Reimer, P. IntCal13 and Marine13 Radiocarbon Age Calibration Curves 0–50,000 Years cal BP. *Radiocarbon* **55,** 1869–1887 (2013).
3. Teklemariam, M., Battaglia, S., Gianelli, G. & Ruggieri, G. Hydrothermal alteration in the Aluto-Langano geothermal field, Ethiopia. *Geothermics* **25,** 679–702 (1996).
4. Hutchison, W. Past, present and future volcanic activity at restless calderas in the Main Ethiopian Rift. (2015). Ph.D Thesis, University of Oxford, 285 pp. (2015).
5. Le Maitre, R. W. *et al. Igneous rocks: a classification and glossary of terms: recommendations of the International Union of Geological Sciences Subcommission on the Systematics of Igneous Rocks*. (Cambridge University Press, 2002).
6. Irvine, T. N. & Baragar, W. R. A. A guide to the chemical classification of the common volcanic rocks. *Can. J. Earth Sci.* **8,** 523–548 (1971).
7. Peccerillo, A. *et al.* Relationships between Mafic and Peralkaline Silicic Magmatism in Continental Rift Settings: a Petrological, Geochemical and Isotopic Study of the Gedemsa Volcano, Central Ethiopian Rift. *J. Petrol.* **44,** 2003–2032 (2003).
8. Giordano, F. *et al.* Genesis and evolution of mafic and felsic magmas at Quaternary volcanoes within the Main Ethiopian Rift: Insights from Gedemsa and Fanta 'Ale complexes. *Lithos* **188,** 130–144 (2014).
9. Mohr, P., Mitchell, J. G. & Raynolds, R. G. H. Quaternary volcanism and faulting at O'A caldera, Central Ethiopian Rift. *Bull. Volcanol.* **43,** 173–189 (1980).
10. Di Paola, G. M. Geology of the Corbetti Caldera area (Main Ethiopian Rift Valley). *Bull. Volcanol.* **35,** 497–506 (1971).
11. Di Paola, G. M. The Ethiopian Rift Valley (between 7° 00′ and 8° 40′ lat. North). *Bull. Volcanol.* **36,** 517–560 (1972).
12. Hutchison, W., Mather, T. A., Pyle, D. M., Biggs, J. & Yirgu, G. Structural controls on fluid pathways in an active rift system: A case study of the Aluto volcanic complex. *Geosphere* 1–21 (2015). doi:10.1130/GES01119.1
13. Yimer, M. The petrogenesis, chemistry and hydrothermal mineralogy of rocks in the Langano-Aluto geothemal system, Ethiopia, Diploma report, Geothermal Institute, University of Auckland, 75 pp. (1984).
14. Mamo, T. Petrography and rock chemistry of well LA-8 Aluto-Langano geothermal system, Ethiopia, Diploma report, Geothermal Institute, University of Auckland, 48 pp. (1985).
15. Klemperer, S. L. & Cash, M. D. Temporal geochemical variation in Ethiopian Lakes Shala, Arenguade, Awasa, and Beseka: Possible environmental impacts from underwater and borehole detonations. *J. African Earth Sci.* **48,** 174–198 (2007).
16. Le Turdu, C. *et al.* The Ziway–Shala lake basin system, Main Ethiopian Rift: Influence of volcanism, tectonics, and climatic forcing on basin formation and sedimentation. *Palaeogeogr. Palaeoclimatol. Palaeoecol.* **150,** 135–177 (1999).
17. Teklemariam, M. Water-rock interaction processes in the Aluto-Langano geothermal field Ethiopia, Ph.D Thesis, University of Pisa, 245 pp. (1996).

18. Rapprich, V. *et al.* Wendo Koshe Pumice: The latest Holocene silicic explosive eruption product of the Corbetti Volcanic System (Southern Ethiopia). *J. Volcanol. Geotherm. Res.* **310,** 159–171 (2016).

19. Lee, J.-Y. *et al.* A redetermination of the isotopic abundances of atmospheric Ar. *Geochim. Cosmochim. Acta* **70,** 4507–4512 (2006).

20. Steiger, R. & Jäger, E. Subcommission on geochronology: convention on the use of decay constants in geo-and cosmochronology. *Earth Planet. Sci. Lett.* **36,** 359–362 (1977).

21. Renne, P. R., Balco, G., Ludwig, K. R., Mundil, R. & Min, K. Response to the comment by WH Schwarz et al. on 'Joint determination of 40 K decay constants and $^{40}Ar^{*}/^{40}K$ for the Fish Canyon sanidine standard, and improved accuracy for $^{40}Ar/^{39}Ar$ geochronology' by PR Renne et al.(2010). *Geochim. Cosmochim. Acta* **75,** 5097–5100 (2011).

22. Renne, P. R. & Norman, E. B. Determination of the half-life of 37 Ar by mass spectrometry. *Phys. Rev. C* **63,** 47302 (2001).

23. Stoenner, R. W., Schaeffer, O. A. & Katcoff, S. Half-lives of argon-37, argon-39, and argon-42. *Science (80-.).* **148,** 1325–1328 (1965).

24. Renne, P. R., Sharp, Z. D. & Heizler, M. T. Cl-derived argon isotope production in the CLICIT facility of OSTR reactor and the effects of the Cl-correction in $^{40}Ar/^{39}Ar$ geochronology. *Chem. Geol.* **255,** 463–466 (2008).

25. Renne, P. R., Knight, K. B., Nomade, S., Leung, K.-N. & Lou, T.-P. Application of deuteron–deuteron (D–D) fusion neutrons to $^{40}Ar/^{39}Ar$ geochronology. *Appl. Radiat. Isot.* **62,** 25–32 (2005).
